# Assistive technology acceptance for visually impaired individuals: a case study of students in Saudi Arabia

**DOI:** 10.7717/peerj-cs.886

**Published:** 2022-03-11

**Authors:** Waleed Al Shehri, Jameel Almalki, Saeed M. Alshahrani, Abdullah Alammari, Faizal Khan, Someah Alangari

**Affiliations:** 1 Department of Computer Science, College of Computer in Al-Leith, Umm Al-Qura University, Saudi Arabia, Al-Leith, Makkah, Saudi Arabia; 2 Department of Computer Science/College of Computing and Information Technology, Shaqra University, Shaqra, Riyadh, Saudi Arabia; 3 Curriculums and Teaching Methods Department, Faculty of Education, Umm Al-Qura University, Makkah, Makkah, Saudi Arabia; 4 Computer Engineering/ College of Computing and Information Technology, Shaqra University, Shaqra, Riyadh, Saudi Arabia; 5 Department of Computer Science, College of Science and Humanities Dawadmi, Shaqra University, Shaqra, Riyadh, Saudi Arabia

**Keywords:** Assistive Technology, Education, Vision, Impaired Individuals, UTAUT, Technology Innovation

## Abstract

Assistive technology (AT) helps students who suffer from visual impairments to achieve their study goals; however, AT’s adoption in Saudi universities is not yet explored. This paper adopts and then extends the Unified Theory of Acceptance and Use of Technology (UTAUT) to incorporate factors influencing the AT’s acceptance based on a designed survey. The survey data was analyzed using Structural Equational Modelling (SEM) with the Partial Least Squares (PLS) technique. The results showed that the factors influencing technology acceptance in this context differed from those previously found to influence acceptance in other contexts. The differences were further studied using post-interview, which shows that the differences are related to limited awareness of visual disability and AT and psychological sensitivity of disabled users in Saudi culture. Moreover, this study provides a list of recommendations for overcoming barriers that limit the acceptance of assistive techniques by Saudi students with visual disabilities. This work’s results provide recommendations for the Saudi government and administrators concerning access to assistive technology in universities and facilitate access to other technologies and other contexts.

## Introduction

In cases where individuals agonize from visual disabilities, they will have difficulties communicating and gaining knowledge; consequently, they could lose social services and education (Al Wadaani et al., 2013). According to the world health organization (Change the definition of blindness, 2017), assistive technology is an immense need for more than 1 billion individuals worldwide, and this number is estimated to be doubled by 2030. The government of Saudi Arabia has adopted Saudi Vision 2030 to transform society and the education system (Saudi Vision 2030, 2018).

The scope of the problem is expanding data on the prevalence of visual impairment had risen to 6% in 2015 (Hersh & Johnson, 2008; Assistive Technology Act, 1998; GaStat, 2017), with 2.6 percent having moderate vision impairment, 2.9 percent having severe vision impairment, and 0.5 percent being blind.

Their many aspects make the AT different in Saudi Arabia; Saudi has cultural differences and customs, thus other family and community attitudes towards disability. Also, Saudi Arabia has different policies relating to the use of AT (Borg & Östergren, 2015; Ahmad, 2015; Davis, Bagozzi & Warshaw, 1992; Pettersson, Appelros & Ahlström, 2007; McDermott, 1993; Cory, 2005) based on organizational administration and structure (Scherer & Galvin, 1996), teachers, experts, and admins who encounter impaired students.

This research addresses the following questions:

RQ1: What variables influence perceptions toward implementing and adopting assistive technologies for vision-impaired students in Saudi universities?

RQ2: To what extent do existing technology acceptance models compensate the acceptance of AT in Saudi universities for vision-impaired students’

RQ3: What can be done to enhance the acceptability of assistive technology at Saudi universities for disabled students?

## Background and Related Work

### Barriers to the use and acceptance of AT

According to Dawe (2006), attitudes about the use of AT can be improperly established, or the user may lack the commitment to master the emerging technologies. As a result, negative views or a lack of excitement for devices can impact how they are used. For example, while AT gadgets are critical for maximizing user mobility, disabled users may negatively feel about them because they rely on them Courtney (2006). Furthermore, based on the specific disability, cultural baggage or stigmas impact the impaired user’s perspectives toward adopting technological assistance (Wanless, 2006; Carlson et al., 2001).

Galvin & Scherer (2004), Scherer (1996), Dorrington et al. (2016) and Edyburn (2015) depicted that users rarely comprehend AT’s role in allowing self-management. People who develop disabilities later in life are often coxswained into using assistive equipment that they later (Löfqvist et al., 2016) observed that more people with disabilities could not complete tasks, prompting caregivers to make choices for them. There is a widespread belief among caretakers and the community that persons with disabilities deserve support regardless of whether or not they use AT, which often leads to users leaving their technical equipment since alternative means of aid are available. To address this issue (Chaurasia, 2016) recommend that the AT experts train both users and caretakers about the device’s ability to change their behavior in parallel. Despite their efforts, one-third of all users quit theirs AT gadgets (Galvin & Scherer, 2004; Holzberg & O’Brien, 2016). This high percentage can be explained by the fact that both users and caretakers have acquired artificial assumptions about the benefits of AT devices. These AT devices fall short of these requirements. Users get dissatisfied and throw the devices away. AT gadgets also have a monetary component that influences how people feel about them. The equipment is costly to purchase, and both the user and caretaker must be trained.

Some gadgets for AT have been built with little regard for the actual limitations of their users. This results in the abandonment of technology (Borg & Östergren, 2015). Manufacturers may not be aware of the real demands and talents of their intended users or aren’t aware of the procedures used to assess the functionality of their technologies. Galvin & Scherer (2004) observed that if the designers create gadgets for a specific objective, that goal must be tailored to suit the needs of the impaired user. To be considered successful, these technologies must be durable, meet the user’s aesthetic standards, be simple to use, and have enough personalization to meet the user’s individual needs. Table 1 lists elements that have been identified as impediments to the adoption of AT in specific situations. The hurdles are divided into three categories, as shown in the table: user issues, teacher constraints, and organizational issues.

### Unified theory of acceptance and use of technology (UTAUT)

**Figure 1:**
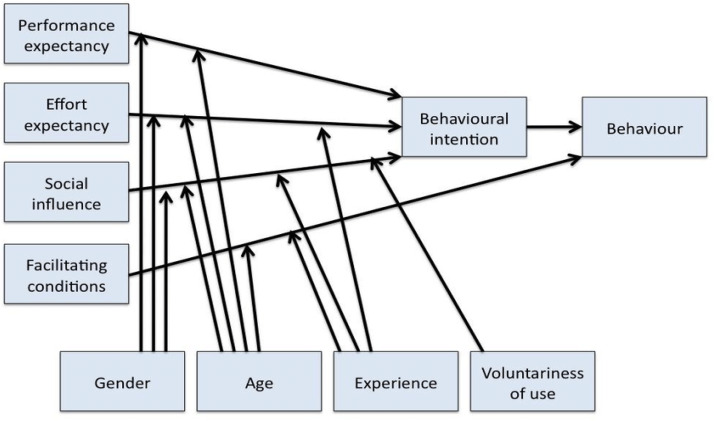
UTAUT model.

The Unified Theory of Acceptance and Use of Technology (UTAUT), developed by Venkatesh & Davis (2000), is a standard paradigm for a generic technology acceptance model. UTAUT, in contrast to previous models, tries to describe both a customer’s intention to use an information system and the user activity that accompanies that purpose. The system was created to provide a full view of the acceptance process than earlier separate models (Venkatesh & Davis, 2000). UTAUT is made up of eight information management models based on psychology, sociology, and telecommunications. Theory of Reasoned Action (TRA), Theory of Planned Behavior (TPB), Davis’s Technology Adoption Model (TAM), TAM2, Motivation Model (MM), Model of PC Utilization (MPCU), Diffusion of Innovation Theory (DOI), and The Social Cognitive Theory (SCT) are some of the examples of these models. While prior models used a variety of factors to model user behavior, UTAUT unifies four independent factors: performance expectancy, effort expectancy, social influence, and facilitating conditions. In addition, four regulating factors such as gender, age, experience, and voluntariness were also included in the model. The UTAUT model is depicted in Fig. 1.

**Table 1:** List of the obstacles to AT use that have been studied in literature.

	User factors				Teacher factors				Institutional factors					
Study	Accessibility	Self-efficacy	Attitude towards AT	Anxiety	Awareness	Time to prepare	Teacher training	Specialists training	Government support	Institutional policies	Pedagogical support	Infrastructure	Cost	Availability
Dorrington, et al. (Hoffman, Sterkenburg & Van Rensburg, 2017)	*												
Borg and Östergren (Cory, 2005)					*								*
Edyburn (Hughes, 2014)		*												
Löfqvist, et al. (Wu et al., 2016)			*										
Orellano-Colón, et al. (McDermott, 1993)													*	*
Chaurasia, et al. (d. F. Alves et al., 2009)				*									
Ahmad (Scherer & Galvin, 1996)							*		*	*			
Holzberg and O’Brien (Shinohara & Wobbrock, 2016)	*								*					
Hoffman, et al. (Abner & Lahm, 2002)				*									
Hughes, et al. (Constantinescu, 2015)	*				*								*
Wu, et al. (Burgos, 2015)			*										
Alves, et al. (Desideri et al., 2016)							*				*	*	
Shinohara and Wobbrock (Bhowmick & Hazarika, 2017)		*											
Abner and Lahm (Venkatesh et al., 2003)							*						
Constantinescu (Davis, Bagozzi & Warshaw, 1989)						*		*	*					*
Burgos (Davis, 1989)								*					
Desideri, et al. (Straub, Keil & Brenner, 1997)	*												*
Bhowmick and Hazarika (Malhotra & Galletta, 1999)			*											
Fakrudeen et al. (Muhammad et al., 2020)		*	*										

The four UTAUT original indicators shown in Table 2 described by Venkatesh & Davis (2000) are as follows The four UTAUT indicators described by Venkatesh & Davis (2000) are as follows:

1. Performance expectancy is an individual’s belief. Applying this to the system would improve the performance of their job.
2. Individual effort expectancy can be easily applied in a system.
3. Social Influence refers to a person’s belief that others’ consideration is necessary to use the system.
4. Facilitating conditions are when a person believes that the organizational and technological infrastructure is in place to facilitate the use of the system.

In the UTAT architecture, performance expectancy (PE) covers elements from prior models such as perceived utility, extrinsic motivation, fitness to the job, facilitating conditions. PE was found to be the strongest predictor of behavioral intention (BI) in a study shown in Venkatesh & Davis (2000), and it was influenced by gender (maximum for male employees) and age (more significant for younger employees), but not by expertise or consent. In the UTUAT model, Effort Expectancy (EE) captures the concepts of perceived usefulness and easy application of complexity. EE was modified by gender (stronger for female workers), age (more significant for older workers), and experience in a validation study (Venkatesh & Davis, 2000). Voluntariness was not recognized as a moderator during the early phases of the system because it was not considered a critical factor.

**Table 2:** The original UTAUT factors.

Factor	Description
Use Behavior (UB)	Describes the user’s actual use of a specific system (Teo et al., 2003), which is dominated by behavioral intention (BI).
Behavioral Intention to Use AT (BI)	Specifies ”the person’s subjective possibility of performing the in question conducts” (Rice & Shook, 1988). BI to use technology has a direct positive impact on usage pattern (Venkatesh & Davis, 2000; Karahanna & Straub, 1999).
Performance Expectancy (PE)	Describes an individual’s assessment of how much they anticipate that the technology will help them to execute tasks better (Venkatesh & Davis, 2000). Components from the DOI theory (Bandura, 1977) are used to calculate PE.
Effort Expectancy (EE)	EE is the degree of adaptability linked to use the technology (Venkatesh & Davis, 2000).
Social Influence (SI)	”The degree to which an individual asses how others believe he or she should use the new system,” (Venkatesh & Davis, 2000)
Facilitating Conditions (FC)	FC defines the degree to which a person perceives that an organization and its technical infrastructure is committed to supporting technology usage (Venkatesh & Davis, 2000)

In the UTAUT paradigm, Social Influence (SI) includes ideas from prior factors, including subjective norm, social variables, and image. It is influenced by a person’s assessment of others’ opinions, the perceived culture of the sample population, individual agreements with other individuals, and the degree of perceived use of innovation to improve one’s social position (Venkatesh & Davis, 2000). The social impact was modified by gender (greater for female employees), age (more for older employees), expertise (more relevant when implementation was in the initial stages), and voluntariness in the testing process. Organizational support, perceived behavioral control, situational factors, and prior model compliance are all favorable conditions for adopting the UTAUT model. FC is portrayed as impacting use behavior rather than behavior intent, unlike earlier models such as TAM. According to the model validation results, FC’s influence on UB was modulated by age (more significant for older employees) and expertise (more resilient during advanced stages of system utilization), but not by gender. Voluntariness was not regarded as a moderator throughout the early stages of the system’s usage. According to Venkatesh & Davis (2000), the UTAUT is responsible for 70% of the variation in acceptance and usage.

The technology adoption model (TAM) (Suebsin & Gerdsri, 2009; Anderson & Schwager, 2004) was created with a wide range of applications in mind, encompassing society (Wills, El-Gayar & Bennett, 2008) and the sociocultural context (Ong et al., 2008). Venkatesh and Davis (Ajzen & Fishbein, 1975) designed TAM 2 as a reaction to TAM. TAM 2 was defined by Suebsin and Gerdsri (Venkatesh & Brown, 2001) as a verification of the previous design. This includes aspects related to cognitive, practical systems and social effects. Venkatesh & Davis (2000) merged all previous variants of the previous design into the unified theory of technology acceptance after adding all the extra elements.

## Conceptual Research Model

Anderson & Schwager (2004), Rogers (2003), Wills, El-Gayar & Bennett (2008), Culnan, (1984) conducted studies where the unified model was successfully employed and verified. We utilize UTAUT because it is one of the most effective hypotheses for explaining differences in technological intention. This could explain 70% of the variance in technology usage compared to the previous theories that could explain only 53% of the variance (Venkatesh & Davis, 2000).

**Figure 2:**
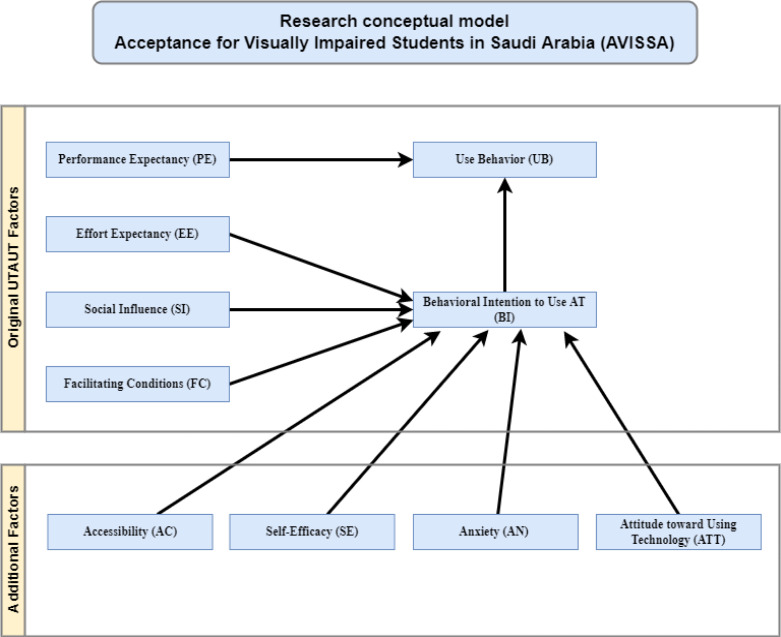
Conceptual research model.

UTAUT, on the other hand, was not used for AT. As a result, this study employs an expanded version of the UTAUT architecture that has been tailored to the study’s needs. Since AT is voluntary at Saudi institutions, the moderating factor voluntariness of use was excluded. Instead, availability, self-efficacy, stress, and perspective towards technology adoption were introduced as additional factors to widen the viewpoint of UTAUT’s on AT adoption. Figure 2 depicts the research’s conceptual model called AVISSA. The additional elements are modeled as impacting the behavioral intent because they are related to the features of the users.

### Original UTAUT factors

#### Additional factors

##### Accessibility (AC).

Physical accessibility refers to a person’s ability to physically utilize the devices and other services such as a computer and internet access as well as AT for visually impaired people. According to Culnan (1984) (Downey, 2006; Hwang & Yi, 2002), the acceptance and use of electronic mail are influenced by physical accessibility (Compeau & Higgins, 1995). Furthermore, Almazroi (2017) claimed that ease of usage also determined the physical accessibility of electronic messaging systems. The findings of these experiments imply that including more information sub-dimensions as separate variables inside our model could provide better meaningful insights for the user activity.

##### Self-Efficacy (SE).

Self-Efficacy (SE) refers to a user’s belief in their ability to complete a specific task (Venkatesh, 2000). SE is a well-known computer behavior prediction that influences a user’s behavioral intent (Asianzu & Maiga, 2012; Colesca, 2009). Researchers and IT experts are interested in SE because of its capacity to encourage end-users, particularly when training and acquiring new skills (Asianzu & Maiga, 2012). Increasing SE levels are expected to result in greater behavioral intention and total IT usage (Rehman, Asif & Ahmad, 2017). Creswell, (2014) discovered that a highly self-assured student’s use of cloud computing in education could increase the implementation of cloud apps. As a result, it is projected that students who are confident in their ability to use AT in their studies would use it more.

##### Anxiety (AN).

Computer apprehension is a significant factor influencing behavioral intention by affecting how easily a technology is being used (Cavana, Delahaye & Sekaran, 2001). As a result, the emotional shame experienced by pupils with computer phobia may lead to a decreased willingness to use technology. In conclusion, this research implies that computer fear will negatively influence students’ intentions of AT usage.

##### Attitude toward Using Technology (ATT).

Venkatesh & Davis (2000) define the perspective towards using technology as an individual’s affective response to the use of a technological system. TAM’s model was developed by Abunadi (2012). They recognized attitudes as a critical component in controlling the link between perceived usefulness, ease of use, and actual behavior. Other dimensions, such as confidence and views regarding safety, were assessed in this study because they were identified as essential contributors (Neuman, 1997; Sekaran, 2003).

Proposed Acceptance for Visually Impaired Students in Saudi Arabia (AVISSA) Factors and Hypotheses, and the Research Approach.

The AVISSA conceptual model and the hypotheses is to be investigated by the model are defined in this section, where “visually impaired student” corresponds to a visually impaired student enrolled in a Saudi institution.

### Factors and hypotheses

Use Behavior (UB): The actual usage of assistive technology by a visually challenged student

Behavioral Intention (BI): The perceptual likelihood of a visually challenged student engaging in AT

Performance Expectancy (PE): the extent to which a visually impaired student trusts that employing AT will help them improve their academic performance.

Effort Expectancy (EE): the degree of comfort with which a visually impaired student can use AT

Social Influence (SI): the extent to which a visually impaired student believes that almost all people are important to him or her, and hence believes that AT should be used in their studies.

Accessibility (AC): the ability of a visually impaired learner to use and utilize AT.

Self-Efficacy (SE): the extent to which a visually impaired student considers that they can complete a task with AT

Anxiety (AN): The positive or negative attitude of a visually impaired student toward using an AT

Attitude Toward Using Technology (ATT): A visually impaired learner’s positive or negative attitude toward utilizing an AT.

They are facilitating conditions (FC): the aspects in the surroundings that make it easier for a visually impaired student to use assistive technology. This is determined by the notion of having access to important resources and the ability to acquire information and the support needed to utilize the AT. Hypotheses of this study is shown in Table 3.

**Table 3:** AVISSA hypotheses.

#	Hypothesis
H1	Performance expectancy (PE) will have a significant favorable influence on behavioral intention to use assistive technologies (BI).
H2	Effort expectancy (EE) will have a significant favorable influence on behavioral intention to use assistive technologies (BI).
H3	Social influence (SI) will significantly influence behavioral intention to use assistive technologies (BI).
H4	Accessibility (AC) will significantly influence behavioral intention to use assistive technologies (BI).
H5	Self-efficacy (SE) will significantly influence behavioral intention to use assistive technologies (BI).
H6	Anxiety (AN) will significantly negatively influence behavioral intention to use assistive technologies (BI).
H7	One’s attitude towards using technology will have a substantial impact on one’s behavioral intention (BI) to utilize AT.
H8	Facilitating conditions (FC) will have a significant favorable influence on use behavior (UB).
H9	Behavioral intention (BI) will have a substantial positive impact on user behavior (UB).

## Research Methodology

### Research approach

**Figure 3:**
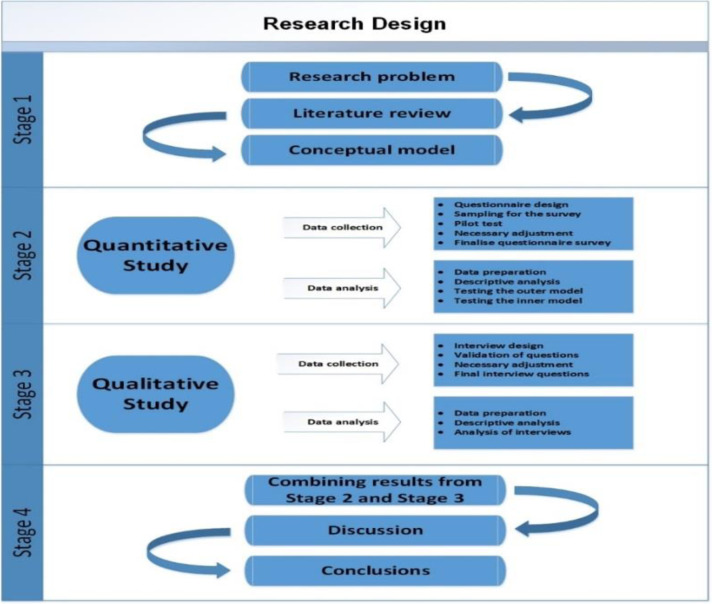
Research design.

This research uses mixed methods that utilize quantitative and qualitative data collection methods (Gill et al., 2008) to arrange objective decision-making alternatives or rules (Green, 1999). Figure 3 depicts the research strategy for this project, which comprises of four stages:

#### Stage 1

The first stage was to construct the study’s issue, which assisted in identifying the deciding elements that might affect the acceptability of AT by visually impaired university students in Saudi Arabia.

#### Stage 2

The first study was a quantitative based. This study collected many survey responses from visually handicapped students in Saudi universities in order to examine the relation between variables of the proposed models. The inclusion criterion was the Saudi participants with least graduation or graduate student between the age of 22 to 50 took part in the survey. The main goal of this stage was to determine the extent to which the factors influenced AT acceptability in Saudi Arabia.

#### Stage 3

A qualitative study was included in Stage 3 to acquire a better understanding of the study outcomes. To check, analyse, explain, and provide a deeper understanding of stage 1 data, a semi-structured interview is conducted by the researcher with Saudi AT users and specialists.

#### Stage 4

Stage 4 entailed gathering, analyzing, and combining the findings from the quantitative and qualitative research in order to provide a clear image and a more simple interpretation of the findings. The results were compared with the previous studies’ results, thus identifying the research community’s contributions to the research community.

### Quantitative study

Quantitative approach strategies are used in positivist research to acquire information from users (Punch, 2013), which results in numerical data to identify related factors that explain the emerging phenomena (Hennink, Hutter & Bailey, 2010).

#### Survey design

The quantitative investigation was conducted using an online survey (checklist) offered by Survey Monkey. Questions were created by Cavana, Delahaye & Sekaran (2001) (Green, 1999) using standards to avoid assessment mistakes. To develop an outcome that can be generalized for an entire society, a 5-point scale was established and random sampling was applied (Gill et al., 2008). There were three parts to the survey. The first section included questions about demography, while the second section focused on the UTAUT model constructs and external influences. The third section allows respondents to leave comments and asked them to get findings by providing their email address if they so desired. As shown in Table 4, data were classified according to item codes and quantitative variables. Positively phrased items were coded with a rating of 5 for “strongly agree” and 1 for “strongly disagree”. Negatively phrased questions (such as item PE5) were coded with “strongly disagree” scoring five points and “strongly agree” scoring one point. For critical examination, the generated data was loaded into IBM-SPSS, and subsequently Smart PLS software PLS-SEM (Chin, 1998) was utilized for advanced analysis. Table 4 lists the measurement items that were altered from prior studies to determine the parameters and sources of this investigation, as well as their codes.

**Table 4:** Measurement items and codes.

Variable	Code	Item	Adapted from
Performance expectancy	PE1	Using Assistive Technology is useful for my study.	(Venkatesh & Davis, 2000; Suebsin & Gerdsri, 2009)
	PE2	Using Assistive Technology enables me to accomplish tasks more quickly.	
	PE3	My productivity improves when I use AT.	
	PE4	If I use Assistive Technology, I will increase my chances of getting a good grade.	
	PE5	It is a waste of time for me to use AT	
	PE6	Using Assistive Technology decreases the time needed for my important study responsibilities.	
Effort Expectancy	EE1	My interaction with Assistive Technology would be clear and understandable.	(Venkatesh & Davis, 2000; Suebsin & Gerdsri, 2009)
	EE2	It would be easy for me to become skillful at using Assistive Technology.	
	EE3	AT would be simple for me to use.	
	EE4	Learning to operate Assistive Technology is easy for me.	
	EE5	I find it easy to use Assistive Technology to get the knowledge that I want.	
	EE6	I find flexibility when dealing with Assistive Technology.	
Social influence	SI1	People who influence my behavior think that I should use Assistive Technology.	(Venkatesh & Davis, 2000)
	SI2	People who are important to me think that I should use Assistive Technology.	
	SI3	The staff of the university has been helpful in the use of Assistive Technology.	
	SI4	In general, the university has supported the use of Assistive Technology.	
	SI5	If my friends used AT, I would use it as well.	
	SI6	My university lecturers are very supportive of the use of AT for my study.	
Facilitating conditions	FC1	I have the necessary resources to use Assistive Technology.	(Venkatesh & Davis, 2000)
	FC2	I have all of the resources I need to use the AT	
	FC3	I am well-versed in the skills required to use the AT	
	FC4	Other systems I use are compatible with the AT	
	FC5	When it comes to AT issues, a specialized person (or group) is available to help.	
	FC6	I’ve had enough experience with AT to be able to use it.	
Attitude toward using technology	ATT1	Assistive Technology appears to be a good fit for my learning style.	(Venkatesh & Davis, 2000)
	ATT2	It is an excellent idea to use assistive technology.	
	ATT3	Studying becomes more pleasurable with the use of AT	
	ATT4	It’s a lot of fun to study with AT	
	ATT5	I enjoy using AT when I’m learning.	
	ATT6	It’s tedious to use AT	
Behavioral intention to use the AT	BI1	Using Assistive Technology is a pleasurable experience.	(Venkatesh & Davis, 2000; Suebsin & Gerdsri, 2009)
	BI2	I intend to make extensive use of AT	
	BI3	In the future, I expect to employ AT	
	BI4	I plan to use Assistive Technology in my study.	
	BI5	I will do my study activities using Assistive Technology.	
Self-efficacy	SE1	I could complete a task using Assistive Technology if there was no one around to tell me what to do as I go.	(Rehman, Asif & Ahmad, 2017; Venkatesh & Davis, 2000)
	SE2	I could complete a task using Assistive Technology to call someone for help if I got stuck.	
	SE3	I could complete a task using Assistive Technology if I had a lot of time to complete it.	
	SE4	I could complete a task using Assistive Technology if I had just the built-in help facility for assistance.	
	SE5	I will be able to overcome many study challenges by using Assistive technology successfully.	
	SE6	I am confident that I can perform effectively on many different tasks by using Assistive Technology.	
	SE7	Compared to other vision impaired students who don’t use Assistive Technology, I can do most tasks very well.	
Anxiety	AN1	I feel apprehensive about using Assistive Technology.	110, (Venkatesh & Davis, 2000)
	AN2	It scares me to think that I could lose a lot of information using Assistive Technology by hitting the wrong key.	
	AN3	I hesitate to use Assistive Technology for fear of making mistakes I cannot correct.	
	AN4	Assistive Technology is somewhat intimidating to me.	
	AN5	I would be reluctant to use Assistive Technology because I’m not too familiar with it.	
Accessibility	AC1	I have easy access to Assistive Technology devices in the university.	(Al-Gahtani, Hubona & Wang, 2007)
	AC2	I will benefit from having easy access to AT equipment around the university.	
	AC3	In order for me to succeed, I need to have AT equipment installed in my classroom.	
	AC4	It is beneficial to have easy access to AT devices at home and at campus.	
	AC5	It will be beneficial to have mobile and portable AT equipment that I may take with me everywhere I go.	
Use behavior	UB1	I’d like to employ AT to help me with my studies.	(Venkatesh & Davis, 2000)
	UB2	I utilize AT on a regular basis.	
	UB3	On a daily basis, I use AT	
	UB4	I used AT for the majority of my academic tasks.	

The online poll was completed by ten visually challenged Saudi students. After that, they were asked if they had any difficulties understanding the survey. Some queries were reworded to improve the understanding as a result of this feedback. Finally, three Saudi Ph.D. students were tasked with determining whether the survey questions accurately assessed each component. Because the majority of Saudis speak Arabic, survey items were translated into Arabic using Sekaran’s recommendations (Murray, 1998).

#### Validity (Pilot Test of the Questionnaire)

In confirmatory observational research, instrument verification is one of the earliest and most important steps (Wills, El-Gayar & Bennett, 2008). Professionals with more knowledge or skill in the field are usually invited to evaluate the survey and comment on whether the scale items used in the study have external validity. The survey was examined in a pilot survey in order to confirm the reliability of the results so that the reliability and clarity of both the items and questions can be confirmed. The online poll was completed by ten visually challenged students from Saudi Arabia. After that, they have been asked if they had any difficulties in understanding the questionnaire. Some queries were rephrased to improve their understanding as a result of this feedback. Furthermore, three PhD students from Saudi Arabia were required to determine whether the survey questions accurately measured each category. After then, changes were made to the tool in order to address the investigators’ concerns.

### Qualitative study

Qualitative methods are a tool that equips researchers with a means to explore specific phenomena to a deeper level (Khan, 2014). According to Alsaghier (2010) most qualitative studies address human behavior and focus on cultural factors that shape human behavior. During this research, qualitative methods were used during stage 3 to confirm, interpret, and explain the quantitative study results to understand the observed behavior. Because qualitative research involves focusing on descriptive data in contrast to statistical data (Hair et al., 2014), this makes it a precious tool in extracting value from questions where ‘why’ and ‘what’ are involved (Sohaib et al., 2019; Nunnally & Bernstein, 1994).

#### Interview design

This research conducted semi-structured interviews with two groups: visually impaired students in Saudi Arabian universities (users) and staff associated with disability support units at Saudi universities (domain experts). Users are very familiar with the specific technology, while domain experts will have a broader perspective and are better placed to address big-picture issues. Following Hair et al. (1998), each interview needed around 45 min to be completed.

## Results

### Normality

In this study, it has been observed that the assessment of the skewness and kurtosis values were within the suggested ranges, as shown in Appendix A. As a result, the data is assumed to be regularly distributed.

### Descriptive statistics

#### Demographic information analysis

We sent the survey questionnaire invitation by email to around 300 visually impaired students in Saudi universities. The respondents were 87 (29%). Three responses were removed due to the presence of outliers. That left *N* = 84 as the dataset entered into SPSS and analyzed. Table 5 gives demographics frequency statistics for the respondents, and the following sections provide the findings of this analysis.

**Table 5:** Demographic characteristics.

Variable		Frequency	Percent
Gender	Male	45	53.6
	Female	39	46.4
Age	18–21	17	20.2
	22–25	34	40.5
	26–29	15	17.9
	30–33	14	16.7
	More than 33	4	4.8
Disability Duration	Since birth	62	73.8
	More than 10 years	15	17.9
	9–5 years	4	4.8
	Less than 5 years	3	3.6
Level of disability	Moderate visual impairment	14	16.7
	Severe visual impairment	37	44
	Blindness	33	39.3
Use of AT	A few times a month	5	6
	A few times a week	8	9.5
	Once a day	2	2.4
	Several times a day	69	82.1
Experience using computers	Beginner	23	27.4
	Intermediate	40	47.6
	Advanced	21	25
Educational level	Diploma degree	7	8.3
	Bachelor degree	60	71.4
	Master degree	15	17.9
	Doctorate	2	2.4
Type of Assistive Technology used	Screen Readers	67	79.8
	Braille Technologies	51	60.7
	Optical Character Recognition	5	6
	Electronic Dictionaries	7	8.3
	Text to Voice Technologies	35	41.7
	Smartphone applications	74	88.1

#### Disability duration

**Figure 4:**
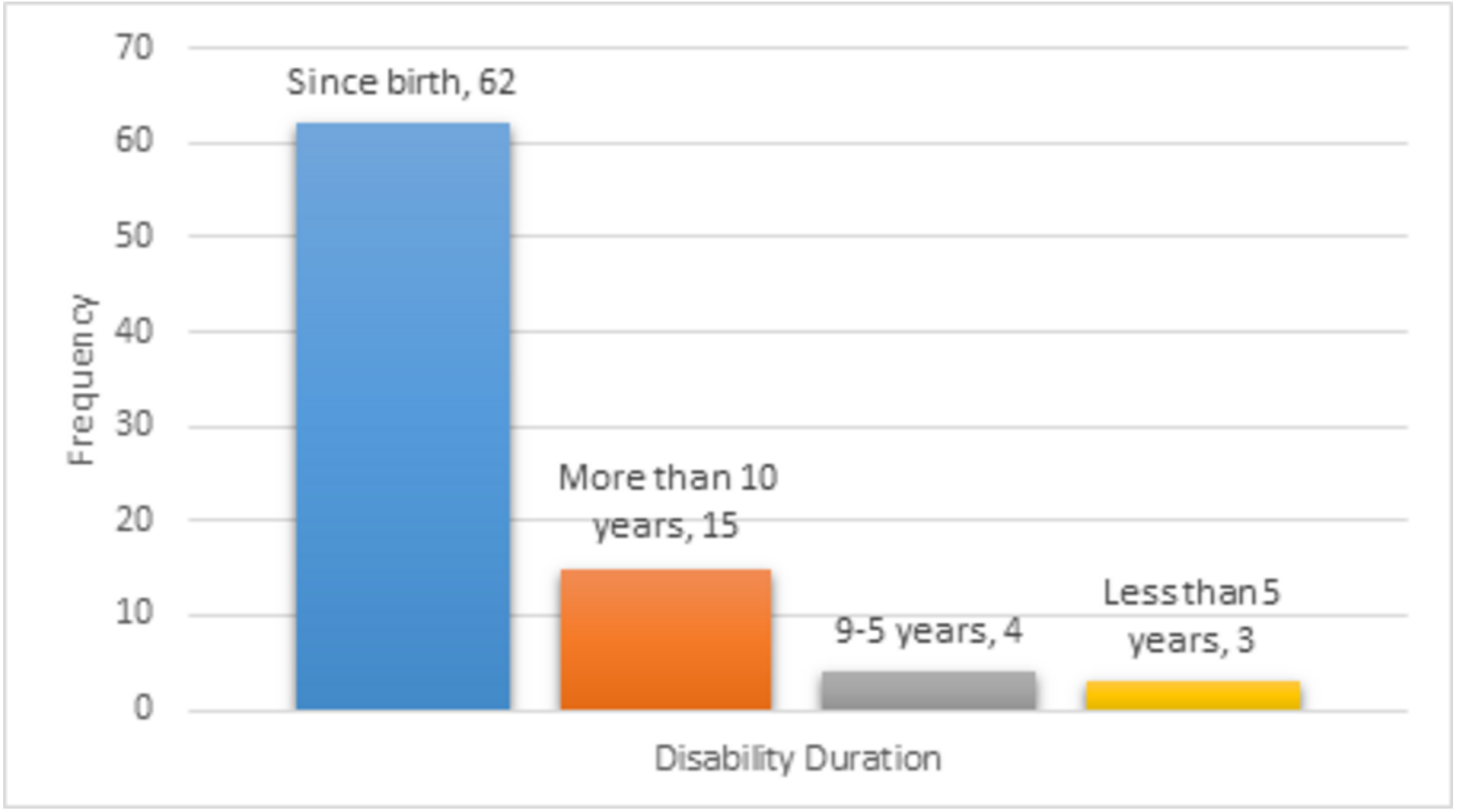
Disability duration of participants.

As Fig. 4 illustrates, most participants have been visually impaired for a relatively long time. The vast majority of respondents, 73.8%, have been visually impaired since birth. Also, a significant minority, around 18%, have been visually impaired for more than ten years.

#### Level of disability

**Figure 5:**
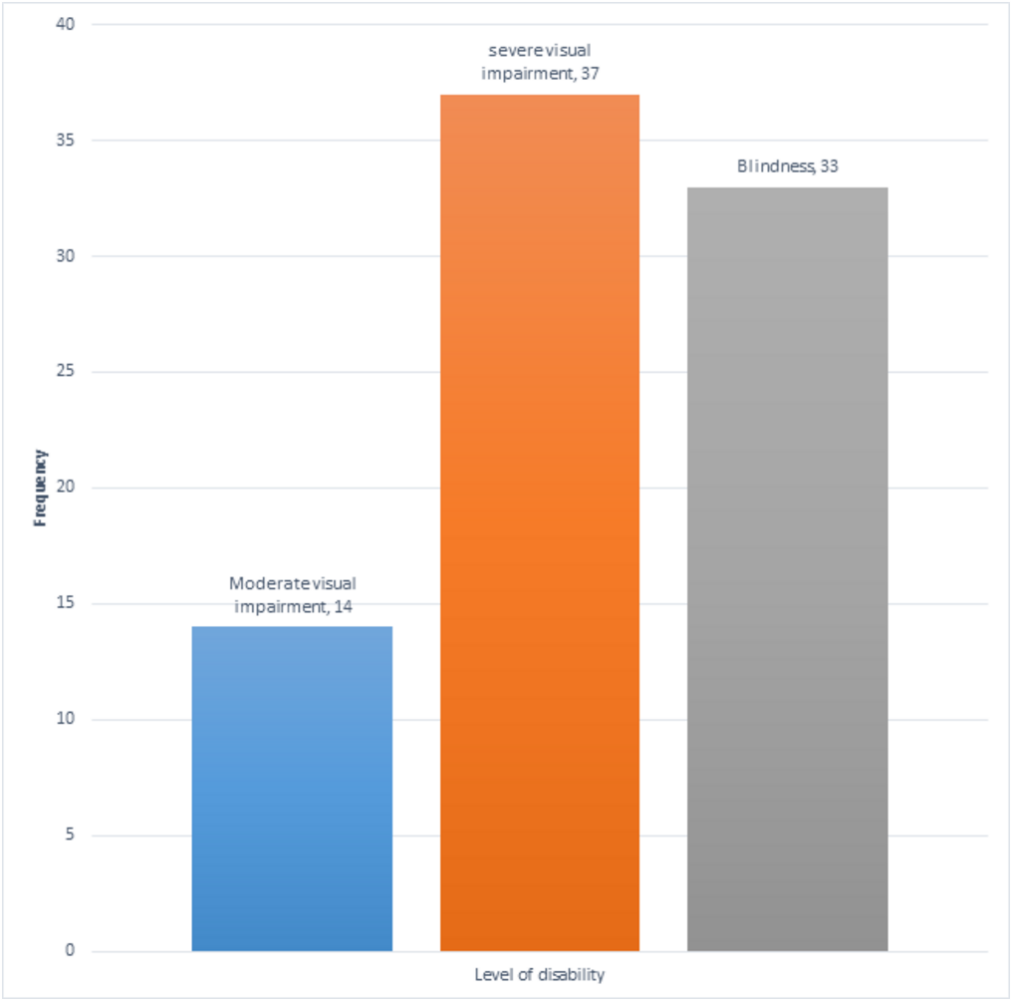
Level of disability of participants.

Figure 5 depicts that 70 (83.3%) participants have severe visual impairment or are blind. Severe visual impairment accounts for 37 (44%) respondents, while 33 (39.3%) are blind.

#### Use of AT

**Figure 6:**
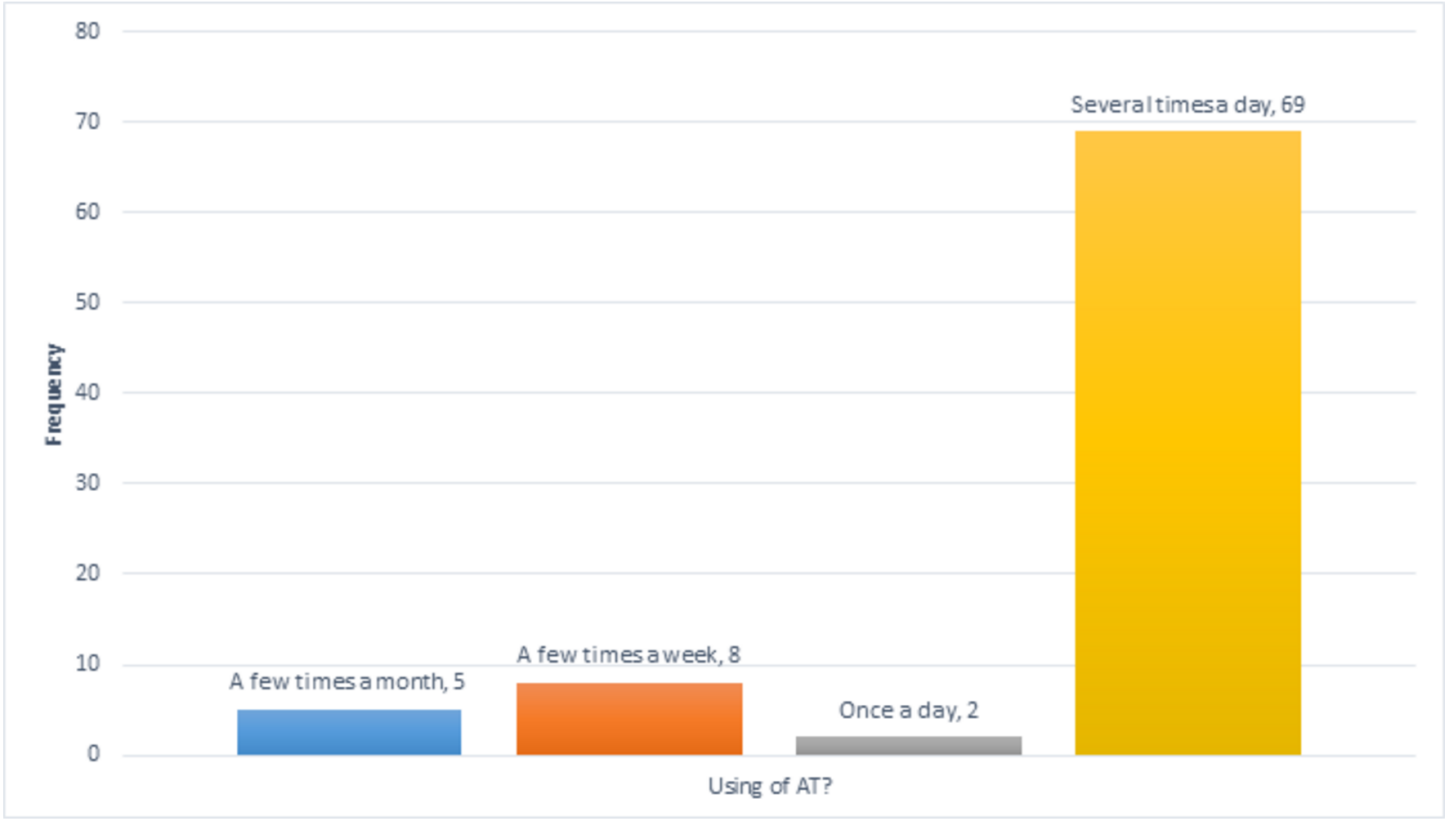
Participants’ experience of using AT.

Figure 6 illustrates that most participants use assistive technology very often, with 69 (82%) doing so several times a day. These results are not surprising since, as we saw earlier, most participants had severe visual impairments or were blind.

#### Type of assistive technology used

**Figure 7:**
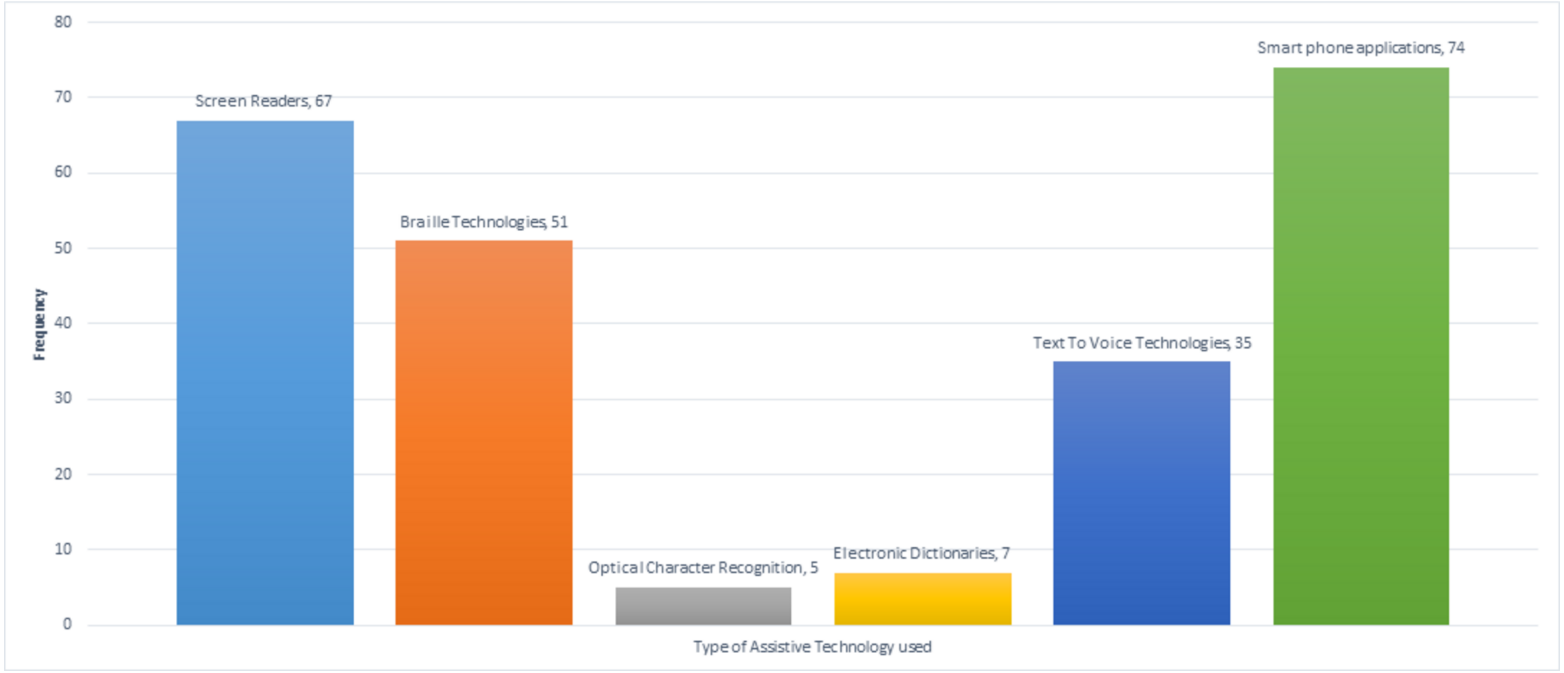
Type of assistive technology used by participants.

Figure 7 show that most respondents (88.1%) use smartphone-based assistive technology. This could be due to smartphones’ widespread use nowadays and because they are comfortable and accessible to use.

### Quantitative data analysis and results

#### Goodness of the measurement model (Outer Model)

Two observational tests were performed: reliability and validity. We used the indicator reliability and internal consistency reliability to assess the proposed model’s adequacy and satisfaction before setting the structural model’s path coefficients (Hair et al., 2016). The loadings and indicators correlations with respective latent variables are used to assess an individual item’s reliability. Loadings for all indicators were computed, and indicators that had a loading less than 0.70 were deleted. As a result of this, 23 indicators were removed, leaving the 32 indicators shown in Table 6

**Table 6:** Indicator reliability.

Indicators	Loading	Reliability (loading^2^)
AC3	0.781	0.610
AC4	0.902	0.814
AC5	0.896	0.803
AN1	0.896	0.804
AN2	0.747	0.557
AN3	0.808	0.653
AN4	0.874	0.763
ATT1	0.754	0.568
ATT2	0.729	0.532
ATT3	0.843	0.711
ATT4	0.864	0.746
BI1	0.741	0.550
BI2	0.836	0.699
BI3	0.839	0.704
BI4	0.882	0.777
EE3	0.724	0.524
EE5	0.899	0.808
EE6	0.907	0.822
FC2	0.801	0.642
FC5	0.852	0.726
FC6	0.877	0.769
PE1	0.812	0.660
PE2	0.816	0.666
PE4	0.805	0.648
SE5	0.865	0.749
SE6	0.885	0.782
SE7	0.646	0.417
SI3	0.830	0.688
SI4	0.993	0.986
UB2	0.897	0.805
UB3	0.885	0.783
UB4	0.817	0.667

Cronbach’s alpha was used to measure internal consistency for study factors regarding the survey sample measurement. Some researchers argue that the acceptable cut-off is 0.7; others claim that any value above 0.6 can be accepted (Emory & Cooper, 1991; Linda, 2018; Gul et al., 2017a). Values shown in Table 7 indicate that both Composite Reliability and Cronbach’s alpha are at acceptable levels. Accordingly, high levels of internal consistency reliability have been exhibited by all reflective latent variables.

**Table 7:** Cronbachs alpha and composite reliability.

Variable	# Items	Cronbach’s alpha	Composite reliability
Accessibility (AC)	**3**	**0.824**	**0.896**
Anxiety (AN)	**4**	**0.865**	**0.900**
Attitude toward using technology (ATT)	**4**	**0.810**	**0.876**
Behavioral intention to use the AT (BI)	**4**	**0.845**	**0.896**
Effort Expectancy (EE)	**3**	**0.810**	**0.883**
Facilitating conditions (FC)	**3**	**0.807**	**0.881**
Performance expectancy (PE)	**3**	**0.743**	**0.852**
Self-efficacy (SE)	**3**	**0.721**	**0.845**
Social influence (SI)	**2**	**0.863**	**0.911**
Use behavior (UB)	**3**	**0.837**	**0.901**
Total	**32**		

### Hypothesized structural model testing

**Figure 8:**
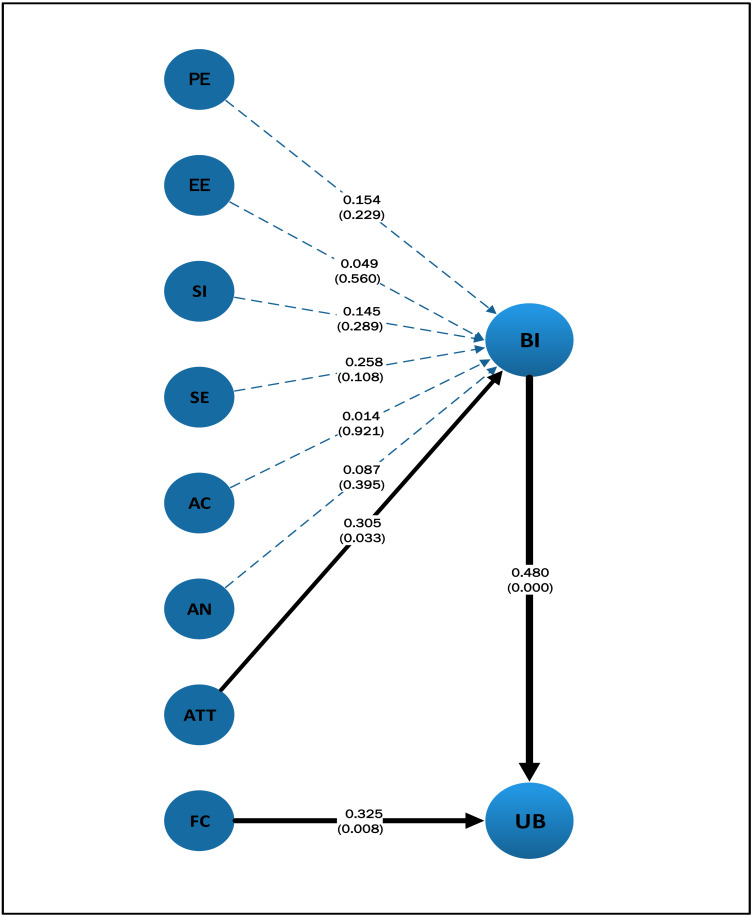
Inner model testing result.

The hypothesis testing aims to identify which independent variables (predictors), together or separately, meaningfully contribute to explaining the dependent variables. The path coefficients and *p*-values (in parentheses) for the relationship between model factors will be discussed in detail in the next section Fig. 8.

Having established that the structural model is a good fit for the data, standardized path coefficients and *p*-values were examined to establish a basis for accepting or rejecting the hypothesized relationships. Summary of inner model testing results shown in Table 8 and the hypothesis associated with each model path. Hypotheses for which the *p*-value of the corresponding path is greater than 0.05 are supported at the 5% confidence level (Al Rub & Al Ahmed, 2014). By this test, the only hypotheses that are supported are H3, H4, and H6. In other words, the effect of BI will be (*p* < 0.05). Moreover, FC (*p* < 0.001) on UB is significant, but the only variable that has a significant effect on BI is ATT (*p* < 0.01). The Hypothesis testing result is shown in Table 8.

### Qualitative data analysis and results

Nine semi-structured interviews were conducted with visually impaired students in Saudi universities (users) and individuals who work in Saudi universities’ disability units and have experience in dealing with visually disabled students (experts). Table 9 shows demographic information for the nine interviewees.

The demographic information shows that interviews were conducted with both users and experts. In this study, Alquraini & Gut (2012) and Al-Gahtani, Hubona & Wang (2007) techniques were merged and used differently to check participants’ reliability and verify the interview. The interviewer read a summary of each interviewee’s interview at the end of the interviews and asked him if that what is he meant. The results’ reliability was ascertained by cross-case analyses, which showed a recurrence of many of the ideas across participants. Data validity was checked by comparing the interview findings with the quantitative findings recommended by Murray (1998). Each interview was transcribed, and then the transcription was compared with the sound recording to confirm that it was free of errors. The transcription was also compared with the interviewer’s hand-written notes during the interview to ensure that the ideas captured in the transcript and notes agreed. This research adhered to Muhammad et al. (2020) four guiding principles used to analyze semi-structured interviews:

Table 10 summarizes the issues suggested by interview participants that may significantly explain why factors investigated in the AVISSA model were not found to affect behavioral intention (BI) significantly. An asterisk in the table indicates that the issue in that row was proposed to explain the interviewee’s indicated acceptance factor in that column.

**Table 8:** Hypothesis testing result.

	Path (hypothesis)	Coefficient	*p*-Value	Hypothesis testing result
H1	**AC ->BI**	0.014	0.921	Not supported
H2	**AN ->BI**	0.087	0.395	Not supported
H3	**ATT ->BI**	0.305	^*^ 0.033	Supported
H4	**BI ->UB**	0.480	^***^ 0.000	Supported
H5	**EE ->BI**	0.049	0.560	Not supported
H6	**FC ->UB**	0.325	^**^ 0.008	Supported
H7	**PE ->BI**	0.154	0.229	Not supported
H8	**SE ->BI**	0.258	0.108	Not supported
H9	**SI ->BI**	0.145	0.289	Not supported

**Notes.**

*** Correlation is Significant at <0.001.

** Correlation is Significant at <0.01.

* Correlation is Significant at <0.05.

**Table 9:** Interviewee demographics.

Participant	User / Expert	Experience with AT
1	User	11 years
2	Expert	5 years
3	User	6 years
4	User	5 years
5	User	8 years
6	Expert	12 years
7	User	6 years
8	Expert	15 years
9	Expert	9 years

**Table 10:** Summary of interviewee opinions.

		Interviewee								
	Group ^∗^	1	2	3	4	5	6	7	8	9
	years	U	E	U	U	U	E	U	E	E
Explanation	Factor	11	5	6	5	8	12	6	15	9
Importance of AT	PE/EE		*	*		*		*		*
No training in AT use	PE/EE				*		*		*	
AT Incompatibility with uni systems	PE/EE					*				
Uni staff unaware of AT	PE/EE						*		*	
Others unfamiliar with AT	SI						*	*	*	*
Psychological sensitivity	SI	*	*	*			*			*
Self-confident and motivated	SE.		*	*	*	*	*	*		*
AT not threatening	AN					*	*	*	*	
AT problems easy to overcome	AN	*			*			*		
Importance of AT	AN	*	*	*						*
Most AT is smartphone-based	AC		*	*	*	*		*	*	*
AT Provided by university	AC.	*			*					

## Discussion

For this study, where the focus is on assistive technology use by visually impaired Saudi students, the UTAUT model was extended to investigate the effect of four additional factors on BI: accessibility (AC), self-efficacy (SE), anxiety (AN), and attitude to technology (ATT). In particular, the quantitative study confirmed that the behavioral intention (BI) of visually impaired Saudi students about the use of assistive technology is influenced by their attitude to technology (ATT), and that their use behavior (UB) is influenced by BI and by facilitating conditions (FC). However, the study found that, in contrast to some previous studies in other domains, there was no significant effect on BI of performance expectancy (PE), effort expectancy (EE), social influence (SI), self-efficacy (SE), accessibility (AC), or anxiety (AN). From the interview discussions, two explanations emerged as to why either PE or EE did not significantly influence BI for the target audience:

• AT is essential to visually impaired students; therefore, they will use it regardless of the expected performance or effort.
• Even though visually impaired students use AT in their daily lives, inadequate support for AT inside some universities means that they cannot use it for their study. Suggested aspects of poor support included lack of training, staff unawareness, and the Incompatibility of university systems with AT. Similarly, interviewees suggested two explanations for why social factors did not significantly influence BI:
• Since most family members and friends of visually impaired students are not themselves visually impaired, they may be unaware of the benefits of AT or the needs of its users.
• Visually impaired university students are often confident in their ability to make decisions for themselves and are therefore less dependent on others’ opinions.

Interviewees were unsurprised that neither self-efficacy nor anxiety significantly affected BI. Because most visually impaired students see the benefit of AT for their daily life and are, therefore, highly motivated to learn how to use it and quickly overcome any anxiety. Finally, interviewees pointed out that since most users in the target audience use smartphone-based AT, accessibility is rarely an issue, which means that a significant BI would be challenging to detect.

### AT Acceptance for visually impaired students in Saudi Arabia

#### Dependence on assistive technology

The relationship between the importance of AT to disabled users and their adoption and use of technology is consistent with a study by Isabelle & Sandrine (2009), which considered the use of technology by school students in Saudi Arabia and found that disabled secondary students use technology more than disabled primary students. The reasons are mainly because secondary students are more familiar with the technology. Although Fakrudeen et al. did not investigate university students explicitly, the comments from interview participants in the current study suggest that students with disabilities at the undergraduate level are even more self-sufficient in their use of technology than are school students, perhaps because of their experience in using the technology over a longer time.

Interview participants thought that AT’s importance to visually disabled students explains why the quantitative study did not find that several UTAUT factors were significant determinants for behavioral intention (BI) for this cohort of users. In particular, they felt that because visually disable students to see AT as essential to their daily life, they are likely to use the technology for their study. They usually adopt AT even if they do not have high expectations of its performance (PE) or if they find that it requires significant effort to use (EE).

Interview participants also suggested that the same effect might explain why there was no significant effect of either self-efficacy (SE) or anxiety (AN) on BI; students will be motivated to master the technology even if it requires considerable commitment on their part, or even if they are initially anxious about the use of the technology.

#### Limited awareness of visual disability

Although most adults are broadly aware of disabilities and many would know of someone who is disabled, unless they are disabled, it is unlikely that most people have a detailed awareness of the needs of disabled people or the importance and benefits of assistive technologies. This lack of understanding is perhaps genuine for visually disabled people because many people take sight for granted and find it difficult to imagine what it might be like for those who lack it. Indeed, it is unlikely that even close friends and immediate family members of visually disabled individuals will fully understand the needs or fully realize AT’s importance and benefits.

Martins, Oliveira & Popovič (2014) found that there is inadequate awareness of how ATs can provide an opportunity for independent living, and Venkatesh & Davis (1996) found that one of the difficulties that hinder students with disabilities is the lack of specialized counseling centers to provide family and friends of disabled students with advice on ATs.

Interviewed participants felt that society’s lack of awareness about the needs of disabled people and lack of knowledge about the importance and benefits of ATs for those with disabilities might make users with visual impairment less inclined to be influenced by people around them, including family and friends. This situation could explain why the quantitative study did not find a significant relationship between behavioral intention (BI) and social influence (SI).

From the interviews, it was clear that most interviewees thought that, concerning technology acceptance in general, social influence *would* be expected to influence behavioral intention. However, for AT acceptance by visually impaired students, they were unsurprised to see that it *was not* significant*.* They pointed out that the social circle for visually impaired students often includes many non-disabled people who are unlikely to have any first-hand AT experience. When considering the use of AT, interviewees felt that visually disabled students would be likely to rely more on the opinions and advice of experts (and, of course, of any in their social circle who are also visually disabled) and less on the views of their wider circle of family and friends.

#### Availability of AT in Saudi Arabian Universities

Discussion with interview participants also identified several problems with the availability of AT in the Saudi context that are likely to affect the adoption of ATs by university students. Although these issues are about availability rather than acceptance, interview participants pointed out that they may have had a secondary effect on the quantitative study results. For example, survey respondents from universities where AT for visual disability was not available (or at least not readily available) may have been unsure how to respond to survey questions about effort expectancy (EE). They may have reasoned: “As the technology is unavailable at my university, then no amount of effort would influence my intention to use it”. Interview participants felt that this effect might have masked the relationship between EE and BI. A similar effect may have masked the relationship between behavioral intention and both performance expectancy (“No matter how good it would be, I cannot use it”) and accessibility (“I have access to the AT, but I cannot use it to get the materials I need”).

Interestingly, interviewees felt that the ready access to AT *via* smartphones (most AT for visual disability is now based on smartphones) might have further contributed to the masking of the relationship between behavioral intention and accessibility: (“I have never had any difficulty accessing the AT, so it is not going to influence whether or not I use it in my study”). The result of easy access to technology afforded by smartphones, particularly in developing countries with limited fixed infrastructure such as power and wired networks, was reported in literature, who investigated the use of phones in education Tanzania. The study found that students feel comfortable using phones in education and believe that phones are the most accessible way to use information technology.

#### System incompatibility with assistive technologies

Interviewees pointed out that the Incompatibility of current ATs with the learning management system in some Saudi universities can result in difficulty using the AT in study activities, limiting the AT’s benefit for students with disabilities. For example, a student with a visual disability may not read educational content through the learning management system or website.

A similar conclusion was reached by Abbad, Morris & De Nahlik (2009). They found that using learning management systems that support access to curricula, such as the Universal Design for Learning (UDL), will encourage visually impaired students to use ATs in education and help teachers create curriculum materials suitable for disabled students. A study by Isabelle & Sandrine (2009) identified a specific problem for Saudi students: there is a lack of AT that is compatible with the Arabic language, and most of the curricula materials in Saudi schools and universities are in Arabic.

### Relationship between explanatory themes and acceptance factors

Table 11 summarizes the previous discussion of the relationships between the themes that emerged from the qualitative study and the AVISSA model factors that the quantitative study investigated as possible determinants of behavioral intention (BI). Specifically, it considers those factors that were expected to influence BI but were found *not* to have a significant effect. For each theme, an asterisk in a particular factor column indicates that interview participants felt that the given theme plays a role in explaining why that factor was found *not* to may not have had a significant effect on behavioral intention (BI) in the context of acceptance of AT by visually disabled Saudi university students.

The table can be read in two ways: to see what factors each theme affects and see which themes affected a particular factor. For example, the table shows that interviewees felt that the importance of AT to visually disabled students contributed to the finding that performance expectancy (PE), effort expectancy (EE), anxiety (AN), and self-efficacy (SE) were not significant in determining behavioral intention (BI). Interviewees felt that both the importance of AT to visually impaired students and the psychological sensitivity of disabled users contributed to the finding that anxiety was not significant in determining BI.

### The effect of context on technology acceptance model factors

Perhaps unsurprisingly, the explanatory themes that emerged from the interviews in the qualitative study are all related to the particular details of the study: the characteristics of the potential users of the technology (visually impaired students), the kind of technology (assistive technology), or the environment of its use (universities in Saudi Arabia). In other words, the information provided by interviewees suggests that the reasons for the difference in findings between this current study and previous studies derive from the context of the study: factors that are significant in some contexts are not significant in others. The conclusion is that technology acceptance models, such as UTAUT, do not apply equally well in all contexts.

The current study is not the first to have reported this context-dependency. Although, several UTAUT-based studies, including those by Motaghian, Hassanzadeh & Moghadam (2013), Ong, Lai & Wang (2004) and Venkatesh & Bala (2008), have confirmed that technology acceptance is significantly affected by performance expectancy (PE), effort expectancy (EE), and social influence (SI), others have found that one or more of the UTAUT factors are not significant in specific contexts. For example, Elasmar & Carter (1996) used UTAUT to investigate technology acceptance in the context of acceptance of knowledge management systems in France and found that PE and EE were not significant behavioral intention determinants. Similarly, Imdad et al. (2007) and Karahanna & Limayem (2000) found that SI was not a significant BI determinant when considering acceptance of internet banking in Portugal.

Table 12 summarizes the findings of technology acceptance studies conducted in a range of contexts, indicating which factors were found to significantly affect behavioral intention (BI) and which were not. Table 12 includes studies that used the original UTAUT model and also the studies that investigated the additional AVISSA factors. For completeness, the table also indicates the findings of the current study and the original UTAUT model. In the table, Y indicates that the factor was found to be significant, N that it was found not to be significant, and that the factor was not investigated in the study. A blank cell indicates that information is not available. The table shows that studies of the same factor in different contexts may reach different conclusions regarding the factor’s significance. For example, Gul et al. (2017b) and Taylor & Todd (1995) found that self-efficacy (SE) had a significant effect on acceptance of E-learning in Jordan.

**Table 11:** The relationship between explanatory themes and model factors.

Theme	PE	EE	SI	AC	AN	SE
Importance of AT		*			*	*
Limited community awareness			*			
Psychological sensitivity					*	*
Availability of AT in universities	*	*		*		

**Table 12:** Significance of factors on BI for technology acceptance studies.

Study	Context	PE	EE	SI	AC	AN	ATT	SE
Current study	AT for students with visual disability in Saudi universities	N	N	N	N	N	Y	N
Original UTAUT by Venkatesh, et al. (Venkatesh & Davis, 2000)	Not specific	Y	Y	Y	–	N	N	N
Al-Gahtani, et al. (Motaghian, Hassanzadeh & Moghadam, 2013)	Cultural effects on organizational IT: Saudi Arabia vs. North America	Y	Y	Y	–	–	–	–
Venkatesh and Zhang (Ong, Lai & Wang, 2004)	Technology acceptance: the US vs. China	Y	Y	Y	–	–	–	–
Chu (Venkatesh & Bala, 2008)	Internet intermediary platforms in China	Y	Y	Y	–	–	–	–
Isabelle and Sandrine (Elasmar & Carter, 1996)	Knowledge management systems in France	N	N	–	–	–	–	–
C. Martins et al. (Imdad et al., 2007)	Internet banking in Portugal	Y	Y	N	–	–	–	–
Venkatesh and Davis (Karahanna & Limayem, 2000)	Not specific	–	–	–	–	–	–	Y
Abbad, et al. (Gul et al., 2017b)	E-learning in Jordan	–	–	–	–	–	–	Y
Davis (Anderson & Schwager, 2004)								Y
Motaghian, et al. (Taylor & Todd, 1995)	Web-based learning systems by Iranian university staff	–	–	–	–	–	–	N
Ong, et al. [101]	E-learning systems by engineers in high-tech companies	–	–	–	–	–	–	N
Venkatesh and Bala [102]		–	–	–	–	Y	–	Y
Elasmar and Carter [103]	E-mail use by university students in the US	–	–	–	–	Y	–	–
Igbaria and Chakrabarti [104]	Business students in the US	–	–	–	–	Y	–	–
Karahanna and Limayem [105]	E-mail use at a financial institution in the US	–	–	–	Y	–	–	–
Toe et al. (Hwang & Yi, 2002)	Not specific	–	–	–	Y	–	–	–
Rice and Shook (Compeau & Higgins, 1995)	Electronic messaging in an aerospace firm	–	–	–	Y	–	–	–
Kafyulilo [106]	Mobile learning in Tanzania	–	–	–	N	–	–	–
Taylor and Todd [107]	IT usage in Canada	–	–	–	–	–	Y	–
Tan and Teo [108]	Internet banking in Singapore	–	–	–	–	–	Y	–
Asianzu and Maiga (Abunadi, 2012)	E-tax services in Uganda	–	–	–	–	–	Y	–
Colesca (Neuman, 1997)	E-government in Romania	–	–	–	–	–	Y	–

Finally, several researchers point out that the significance of some technology acceptance factors changes over time, so that factors that played a larger role in the past may now be less important.

## Conclusion

This research is based on the Unified Theory of Acceptance and Use of Technology (UTAUT), using an expanded model that incorporates factors that has previously been important in AT use. According to the original UTAUT model, acceptance is influenced by performance expectancy (PE), effort expectancy (EE), social influence (SI), and facilitating conditions (FC). This research also considered access (AC), self-efficacy (SE), anxiety (AN), and attitude to technology (ATT). Analysis of data from a survey of visually impaired students in Saudi universities showed that only one of the original UTAUT factors (FC) and only one of the additional factors (ATT) had a significant effect on AT acceptance. The survey results were analyzed using structural equation modeling with the partial least-squares technique (PLS-SEM). A follow-up study was conducted using semi-structured interviews of users (visually impaired students) and experts (workers in disability support units) to seek explanations for the differences between these results and those obtained in other contexts. Interviewees suggested several context-specific reasons why acceptance factors may be different for assistive technology (rather than other technologies), for university students (rather than other demographics), or Saudis (rather than citizens of other countries). We summarize results as follows: (1) The importance of AT in the daily activities of visually disabled users may incline users to overlook problems with performance, ease of use, anxiety, and self-efficacy, (2) Limited community awareness of disabilities and assistive technologies in Saudi Arabia may lead users to discount the opinions of friends and family, and (3) Disabled users in Saudi culture may be sensitivity to perceptions of pity, which may result in a determination to be self-reliant in making decisions about their disability.

### Contributions of the study

This study makes contributions in three areas:

• It has facilitated the development of technology acceptance models by enhancing the UTAUT paradigm in order to include assistive technology in education.
• It has aided technological acceptability studies by applying the extended model to the test in a real-world setting.
• It has aided the Saudi educational system by examining the elements that influence of accepting the assistive technology among visually impaired Saudi university students.

All of the features of the original UTAUT model were included in the extended UTAUT model (AVISSA). Moreover, additional factors discovered in the literature were used as an influencing assistive technology adoption. In combination with the AVISSA paradigm, a new survey tool was created to collect data on user opinions towards the acceptance of assistive technology. The new instrument is based on the old UTAUT instrument, with the phrase changed in order to expressly refer to assistive technology in an educational context. Moreover, new questions were also added for each of the extra acceptance factors. Both the AVISSA model and survey instrument were evaluated in the framework in order to investigate the factors which were affecting the acceptability of assistive technology to be used in Saudi institutions. The inquiry was prompted by a lower-than-expected AT uptake among Saudi university students. The need for these strategies was to overcome the overall obstacles present in the system. Data was collected by an online survey among the visually impaired students in Saudi universities, and the survey instrument’s validity was confirmed through a pilot test.

The results of the survey were analysed using partial least-squares structural equation method (PLS-SEM). Based on the survey results, it can be observed that only one ne of the original ATUAT characteristics and one of the extra AT-specific factors had a substantial effect on accepting the research environment. Structured interviews with AT users and AT support personnel were done in order to follow up the findings. The interviews revealed that the study-specific characteristics that explain the variations between the results and those from previous UTAUT-based studies. A detailed survey data and analysis were shown in Chapter 6. The results of the follow-up interviews are presented in Chapter 7, and Chapter 8 explores how the issues raised in the interviews connect to the model factors.

### Recommendations of this study

This section outlines the suggestions that results from the study’s contributions to the scientific community.

Recommendations for the Saudi Government

• The Saudi government must focus on establishing infrastructure to enable the digital conduct of e-governance and e-learning in order to accomplish the digital transformation of Saudi society that is at the heart of Saudi Vision 2030. (Saudi Vision 2030, 2018). In order to access to these service for disabled Saudis, government, educational systems and their websites must be compatible with assistive technology.

Recommendations for Saudi Universities

• Universities in Saudi Arabia should provide improved support for impaired students, including a learning environment that is tailored to their needs and adequate infrastructure. In order to encourage the continuing study, all Saudi universities should establish specialized handicap support sections.
• Disabled students should be taught how to use assistive technologies in the classroom of Saudi universities, thereby expanding their opportunity to use these tools.

## Supplemental Information

10.7717/peerj-cs.886/supp-1 Supplemental Information 1 Raw data

10.7717/peerj-cs.886/supp-2 Supplemental Information 2 Final survey for Quantitative part

10.7717/peerj-cs.886/supp-3 Supplemental Information 3 Final survey for Qualitaive part

10.7717/peerj-cs.886/supp-4 Supplemental Information 4 Arabic Survey

10.7717/peerj-cs.886/supp-5 Supplemental Information 5 Arabic Interview Questions

10.7717/peerj-cs.886/supp-6 Supplemental Information 6

## Additional Information and Declarations

### Data Availability

The following information was supplied regarding data availability: The raw data is available in the Supplementary File and the coding of the data is available in Table 4.
