# Supplementary material for: Assistive technology acceptance for visually impaired individuals: a case study of students in Saudi Arabia
Source: PeerJ Comput Sci. 2022 Mar 11;8:e886. doi: 10.7717/peerj-cs.886 (PMC9044340; doi:10.7717/peerj-cs.886)
Supplement: Supplemental Information 2 [file peerj-cs-08-886-s002.pdf]

Survey

(Assistive Technology is: Any item, piece of equipment, or product system, whether acquired commercially off the shelf, modified, or customized, that is used to increase, maintain, or improve functional capabilities of individuals with disabilities)

Part one. **Citizen demography**: Please choose the most appropriate answer for the following items.

Q1) Gender

- ○ Male
- ○ Female

Q2)) What is your age?

- ○ 18-21
- ○ 22–25
- ○ 26–30
- ○ 31–34
- ○ Above 34

Q3) What is your current education level?

- ○ Diploma degree
- ○ Bachelor degree
- ○ Master degree
- ○ Doctorate or higher

Q4) How would you rate your computer skills?

- ○ Beginner
- ○ Intermediate
- ○ Advance

Q5) How often do you use Assistive Technology?

- ○ Once a month
- ○ A few times a month
- ○ A few times a week
- ○ Once a day
- ○ Several times a day

Q6) How long have you been visually impaired?

- ○ Since birth
- ○ More than 10 years
- ○ 10-5 years
- ○ Less than 5 years

Q7) What is your level of vision impairment?

- ○ Moderate visual impairment
- ○ severe visual impairment
- ○ Blindness

Q8) What is your university name?

- ○ King Saud
- ○ King Abdulaziz
- ○ King Fisal
- Other ………………

Q9) Select the type of Assistive Technology that you normally use. *(You can choose more than one).*

- ○ Screen Readers
- ○ Braille Technologies
- ○ Optical Character Recognition
- ○ Electronic Dictionaries
- ○ Text Windows
- ○ Text Telephones
- ○ Smart phone applications
- ○ Others ………………

Part two. **Performance Expectancy:** Please choose how much you agree with each of the statements within your general experience with Assistive Technology.					
**Item**	**Strongly Disagree**	**Disagree**	**Neutral**	**Agree**	**Strongly Agree**
Using Assistive Technology is useful for my study.					
Using Assistive Technology enables me to accomplish tasks more quickly.					
Using Assistive Technology increases my productivity.					
If I use Assistive Technology, I will increase my chances of getting a good grade.					
Using Assistive Technology wastes my time.					
Using Assistive Technology decreases the time needed for my important study responsibilities.					

***Part three*. Effort Expectancy:** Please choose the degree to which you believe that using Assistive Technology would be free of effort.					
**Item**	**Strongly Disagree**	**Disagree**	**Neutral**	**Agree**	**Strongly Agree**
My interaction with Assistive Technology would be clear and understandable.					
It would be easy for me to become skilful at using Assistive Technology.					
I would find Assistive Technology easy to use.					
Learning to operate Assistive Technology is easy for me.					
I find it easy to use Assistive Technology to get the knowledge that I want.					
I find flexibility when dealing with assistive technology.					

Part four. **Social Influence:** Please choose the degree to which you agree with the following statements.					
**Item**	**Strongly Disagree**	**Disagree**	**Neutral**	**Agree**	**Strongly Agree**
People who influence my behaviour think that I should use Assistive Technology.					
People who are important to me think that I should use Assistive Technology.					
The staff of the university have been helpful in the use of Assistive Technology.					
In general, the university has supported the use of Assistive Technology.					

I would use Assistive Technology if my friends used them.					
The university lecturers are very supportive of the use of Assistive Technology for my study.					

Part five. **Facilitating Conditions:** Please choose the degree to which you agree with the following statements.

Item	Strongly Disagree	Disagree	Neutral	Agree	Strongly Agree
I have the necessary resources to use Assistive Technology.					
I have the necessary knowledge to use Assistive Technology.					
Assistive Technology is compatible with other systems I use.					
A specific person (or group) is available for assistance with Assistive Technology difficulties.					
I have enough experience to use Assistive Technology.					
I think that Assistive Technology fits well with my learning style.					

Part six. **Attitude toward using technology:** Please choose the degree to which you agree with the following statements.

Item	Strongly Disagree	Disagree	Neutral	Agree	Strongly Agree
Using Assistive Technology is a good idea.					
Assistive Technology makes study more interesting.					
Studying with Assistive Technology is fun.					
I like studying with Assistive Technology.					
Using Assistive Technology is boring.					
Using Assistive Technology is pleasant.					

Part seven. **Behavioural intention to use the AT:** Please choose the degree to which you agree with the following statements.

Item	Strongly Disagree	Disagree	Neutral	Agree	Strongly Agree
I intend to use Assistive Technology frequently.					
I predict that I will use Assistive Technology in the future.					
I predict I will continue to use Assistive Technology on a regular basis.					
I plan to use Assistive Technology in my study.					

I will do my study activities using Assistive Technology.					

Part eight. Self-efficacy: Please choose the degree to which you agree with the following statements.

Item	Strongly Disagree	Disagree	Neutral	Agree	Strongly Agree
I could complete a task using Assistive Technology if there was no one around to tell me what to do as I go.					
I could complete a task using Assistive Technology if I could call someone for help if I got stuck.					
I could complete a task using Assistive Technology if I had a lot of time to complete it.					
I could complete a task using Assistive Technology if I had just the built-in help facility for assistance.					
I will be able to successfully overcome many study challenges by using Assistive technology.					
I am confident that I can perform effectively on many different tasks by using Assistive Technology.					
Compared to other vision impaired students who don't use Assistive Technology, I can do most tasks very well.					

Part nine. Anxiety: Please choose the degree to which you agree with the following statements.

Item	Strongly Disagree	Disagree	Neutral	Agree	Strongly Agree
I feel apprehensive about using Assistive Technology.					
It scares me to think that I could lose a lot of information using Assistive Technology by hitting the wrong key.					
I hesitate to use Assistive Technology for fear of making mistakes I cannot correct.					
Assistive Technology is somewhat intimidating to me.					
I would be reluctant to use Assistive Technology because I'm not too familiar with it.					

Part ten. Accessibility: Please choose the degree to which you agree with the following statements.

Item	Strongly Disagree	Disagree	Neutral	Agree	Strongly Agree
I have easy access to Assistive Technology devices in the university.					
Easy access to Assistive Technology devices in many locations in the university will help me.					
The installation of Assistive Technology devices in my classroom is important for my success.					
Easy access to Assistive Technology devices at home and university is helpful.					

Mobile and portable Assistive Technology devices that I can bring anywhere will be useful.					

Part eleven. **Use behaviour:** Please choose the degree to which you agree with the following statements.

Item	Strongly Disagree	Disagree	Neutral	Agree	Strongly Agree
I want to use Assistive Technology to perform my study activities.					
I frequently use Assistive Technology.					
I use Assistive Technology on a regular basis.					
Most of my study tasks were done using Assistive Technology.					

Thank you very much for your time and valuable contribution to this research.

- If you have any further comment or suggestion, please include it in the following space:

……
……
……
……
……

- If you are interested in receiving the results of this study or participating further, please add your email.

……
……
………
