# Supplementary material for: Assistive technology acceptance for visually impaired individuals: a case study of students in Saudi Arabia
Source: PeerJ Comput Sci. 2022 Mar 11;8:e886. doi: 10.7717/peerj-cs.886 (PMC9044340; doi:10.7717/peerj-cs.886)
Supplement: Supplemental Information 3 [file peerj-cs-08-886-s003.pdf]

Interview Questions

This interview seeks your views of the results of a survey about attitudes of visually impaired students towards acceptance and use of assistive technology (AT). Before participating in the interview, please carefully read the document entitled Quantitative Report, which provides a background to the project, the research methodology, and the results of the survey.

Your details

- What university do you study in / work to?
- What is your position?
- How would you likely describe your experience with AT?
- What types of AT you use / deal with?

Your views

Category 1: Performance expectancy

- How does 'expecting good performance' affect the 'intention to use AT? Why?
- How do you think that age will affect this relationship? Please elaborate?
- How do you think that gender will affect this relationship? Why?
- How do you think that experience with computers will affect this relationship? Why?
- How do you consider that level of education will affect this relationship? Please elaborate?
- How do you think the duration that an individual has had a visual impairment will affect this relationship? Please elaborate?
- How do you think the severity of visual impairment will affect this relationship? Please elaborate?

Category 2: Effort expectancy

- How does 'expecting less effort when using AT' affect the 'intention to use it? Please elaborate?
- How do you think that age will affect this relationship? Please elaborate?
- How do you think that gender will affect this relationship? Why?
- How do you think that experience with computers will affect this relationship? Why?
- How do you consider that level of education will affect this relationship? Please elaborate?
- How do you think the duration that an individual has had a visual impairment will affect this relationship? Please elaborate?

- How do you think the severity of visual impairment will affect this relationship? Please elaborate?

Category 3: Social influence

- How does social influence affect the 'intention to use AT? Why?
- How do you think that age will affect this relationship? Please elaborate?
- How do you think that gender will affect this relationship? Why?
- How do you think that experience with computers will affect this relationship? Why?
- How do you consider that level of education will affect this relationship? Please elaborate?
- How do you think the duration that an individual has had a visual impairment will affect this relationship? Please elaborate?
- How do you think the severity of visual impairment will affect this relationship? Please elaborate?

Category 4: Accessibility

- How does the degree to which a person has the ability to access and use AT can affect the 'intention to use AT? Please elaborate?
- How do you think that age will affect this relationship? Please elaborate?
- How do you think that gender will affect this relationship? Why?
- How do you think that experience with computers will affect this relationship? Why?
- How do you consider that level of education will affect this relationship? Please elaborate?
- How do you think the duration that an individual has had a visual impairment will affect this relationship? Please elaborate?
- How do you think the severity of visual impairment will affect this relationship? Please elaborate?

Category 5: Self-efficacy

- How does increasing a user's ability to perform a specific task by using AT can affect the 'intention to use it? Please elaborate?
- How do you think that age will affect this relationship? Please elaborate?
- How do you think that gender will affect this relationship? Why?
- How do you think that experience with computers will affect this relationship? Why?
- How do you consider that level of education will affect this relationship? Please elaborate?
- How do you think the duration that an individual has had a visual impairment will affect this relationship? Please elaborate?

- How do you think the severity of visual impairment will affect this relationship? Please elaborate?

Category 6: Anxiety

- How does anxiety about using AT can affect a user's intention to use it? Why?
- How do you think that age will affect this relationship? Please elaborate?
- How do you think that gender will affect this relationship? Why?
- How do you think that experience with computers will affect this relationship? Why?
- How do you consider that level of education will affect this relationship? Please elaborate?
- How do you think the duration that an individual has had a visual impairment will affect this relationship? Please elaborate?
- How do you think the severity of visual impairment will affect this relationship? Please elaborate?

Category 7: Attitude toward using technology

- How does the user's attitude towards technology can affect the behavioural intention to use AT? Please elaborate?
- How do you think that age will affect this relationship? Please elaborate?
- How do you think that gender will affect this relationship? Why?
- How do you think that experience with computers will affect this relationship? Why?
- How do you consider that level of education will affect this relationship? Please elaborate?
- How do you think the duration that an individual has had a visual impairment will affect this relationship? Please elaborate?
- How do you think the severity of visual impairment will affect this relationship? Please elaborate?

Category 8: Behavioural intention to use AT

- How does the intention to use AT can affect actual user behaviour? Why?
- How do you think that age will affect this relationship? Please elaborate?
- How do you think that gender will affect this relationship? Why?
- How do you think that experience with computers will affect this relationship? Why?
- How do you consider that level of education will affect this relationship? Please elaborate?
- How do you think the duration that an individual has had a visual impairment will affect this relationship? Please elaborate?

- How do you think the severity of visual impairment will affect this relationship? Please elaborate?

Category 9: Facilitating conditions

- How does facilitating conditions such as obtaining resources and knowledge necessary to use AT can affect actual user behaviour? Please elaborate?
- How do you think that age will affect this relationship? Please elaborate?
- How do you think that gender will affect this relationship? Why?
- How do you think that experience with computers will affect this relationship? Why?
- How do you consider that level of education will affect this relationship? Please elaborate?
- How do you think the duration that an individual has had a visual impairment will affect this relationship? Please elaborate?
- How do you think the severity of visual impairment will affect this relationship? Please elaborate?

Category 10: additional information

- Please choose the most important factors of the following factors to indicate your view of its effect on the user's acceptance of AT.

  …… Performance expectancy

  …… Effort expectancy

  …… Attitude toward using technology

  …… Social influence

  …… Facilitating conditions

  …… Self-efficacy

  …… Anxiety

  …… Accessibility
- Why do think these factors are very important?
- What other factors do you believe can affect a user's acceptance of assistive technology?
- Do you have any further comment or suggestion?

Thank you very much for your time and valuable contribution to this research.
