# Supplementary material for: Assistive technology acceptance for visually impaired individuals: a case study of students in Saudi Arabia
Source: PeerJ Comput Sci. 2022 Mar 11;8:e886. doi: 10.7717/peerj-cs.886 (PMC9044340; doi:10.7717/peerj-cs.886)
Supplement: Supplemental Information 4 [file peerj-cs-08-886-s004.pdf]

الجزء الأول : المعلومات العامة

الرجاء اختيار ماتراه مناسبا لك من العناصر التالية

س1) الجنس

- انثى
- ذكر

س2) حدد عمرك من التالي :

- 18-21
- 22-25
- 26-30
- 31-34
- أكبر من 34

س3 ما هو المؤهل التعليمي الذي تقوم بدراسته حالياً ؟

- دبلوم
- بكالوريوس
- ماجستير
- دكتوراه

س4) كيف تقيم نفسك في مهارات استخدام الحاسب الآلي ؟

- مبتدئ
- متوسط
- متقدم

س5) منذ متى وأنت تعاني من إعاقة بصرية ؟

- منذ الولادة
- منذ أكثر من 10 سنوات
- من 5 الى 10 سنوات
- أقل من 5 سنوات

س6) ماهو مستوى الإعاقة البصرية لديك ؟

- ضعف بصر معتدل
- ضعف بصر شديد
- عمى كلي

س8) ماهي الجامعة التي تدرس بها حالياً ؟

- الملك سعود
- الملك عبدالعزيز
- الملك فيصل
- أخرى حدد ....

س 9) اختر نوع التقنية المساعدة التي تستخدمها عادةً (يمكنك اختيار اكثر من واحدة) ؟

- قارئات الشاشة
- تقنيات التعرف الضوئي على الحروف
- تقنيات تحويل النص إلى صوت
- أخرى.......................................
- تقنيات بريل
- القواميس إلكترونية
- تطبيقات الهواتف الذكية

الجزء الثاني : الأداء المتوقع

الرجاء اختيار ما يعبر عن مدى موافقتك على كل عبارة من العبارات التالية :

العنصر	لا أوافق بشدة	لا أوافق	محايد	أوافق	أوافق بشدة
استخدام التقنيات المساعدة مفيد لي في دراستي					
استخدام التقنيات المساعدة يسمح لي أن انجز مهامي الدراسية بشكل أسرع					
استخدام التقنيات المساعدة يزيد من انتاجي					
إذا استخدمت التقنيات المساعدة سوف أزيد من فرصي في الحصول على معدل دراسي أعلى					
استخدام التقنيات المساعدة يضيع وقتي					
استخدام التقنيات المساعدة يساعد في تقليص الوقت المستهلك في الواجبات الدراسية الهامة					

الجزء الثالث : الجهد المتوقع

الرجاء اختيار ما يعبر عن مدى موافقتك على كل عبارة من العبارات التالية :

العنصر	لا أوافق بشدة	لا أوافق	محايد	أوافق	أوافق بشدة
تفاعلي مع التقنيات المساعدة واضح ومفهوم					
اجد من السهل بالنسبة لي ان اصبح متقن لاستخدام التقنيات المساعدة					
أجد أن التقنيات المساعدة سهلة الاستخدام					
تعلم كيفية استخدام التقنيات المساعدة سهل بالنسبة لي					
أجد من السهل استخدام التقنيات المساعدة للحصول على المعرفة التي أريد					
اجد مرونة عند تعاملي مع التقنيات المساعدة					

الجزء الرابع : التأثير الإجتماعى.

الرجاء اختيار ما يعبر عن مدى موافقتك على كل عبارة من العبارات التالية :

العنصر	لا أوافق بشدة	لا أوافق	محايد	أوافق	أوافق بشدة
يعتقد الناس الذين لهم تأثير على سلوكي بأنه يجب علي استخدام التقنيات المساعدة					
يعتقد الناس المهمين بالنسبة لي انه يجب علي استخدام التقنيات المساعدة					
يساعدني موظفي الجامعة في استخدام التقنيات المساعدة					
بشكل عام تدعم الجامعة استخدام التقنيات المساعدة					
سوف استخدم التقنيات المساعدة إذا استخدمها أصدقائي					
يؤيد أعضاء هيئة التدريس بالجامعة استخدام التقنيات المساعدة في الدراسة					

الجزء الخامس : الحالات الميسرة.

الرجاء اختيار ما يعبر عن مدى موافقتك على كل عبارة من العبارات التالية :

العنصر	لا أوافق بشدة	لا أوافق	محايد	أوافق	أوافق بشدة
لدي الموارد اللازمة لاستخدام التقنيات المساعدة					
لدي المعلومات والمعرفة اللازمة التي تجعلني استخدم التقنيات المساعدة					
التقنيات المساعدة متوافقة مع الأنظمة الأخرى التي استخدامها.					
يوجد شخص (أو مجموعة أشخاص) بالجامعة للمساعدة في صعوبات استخدام التقنيات المساعدة					
لدي الخبرة الكافية لاستخدام التقنيات المساعدة					

أعتقد بأن استخدام التقنيات المساعدة يتناسب تماما مع طريقة التعلم الخاصة بي					

الجزء السادس : المواقف تجاه استخدام التكنولوجيا.

الرجاء اختيار ما يعبر عن مدى موافقتك على كل عبارة من العبارات التالية :

العنصر	لا أوافق بشدة	لا أوافق	محايد	أوافق	أوافق بشدة
استخدام التقنيات المساعدة فكرة جيدة					
استخدم التقنيات المساعدة يجعل التعلم اكثر إثارة					
التعليم باستخدام التقنيات المساعدة ممتع					
يعجبني التعليم باستخدام التقنيات المساعدة					
استخدام التقنيات المساعدة ممل					
استخدام التقنيات المساعدة مسلي					

الجزء السابع : النية السلوكية.

الرجاء اختيار ما يعبر عن مدى موافقتك على كل عبارة من العبارات التالية :

العنصر	لا أوافق بشدة	لا أوافق	محايد	أوافق	أوافق بشدة
إنني انوي استخدام التقنيات المساعدة في كثير من الاحيان					
أتوقع أنني يجب أن استخدم التقنيات المساعدة في المستقبل					
أتوقع أنني سوف أستمر في استخدام التقنيات المساعدة بشكل منتظم					
أنا أخطط لاستخدام التقنيات المساعدة في دراستي					
أود أن أؤدي أنشطتي الدراسية باستخدام التقنيات المساعدة					

الجزء الثامن : الكفاءة الذاتية.

الرجاء اختيار ما يعبر عن مدى موافقتك على كل عبارة من العبارات التالية :

العنصر	لا أوافق بشدة	لا أوافق	محايد	أوافق	أوافق بشدة
يمكنني اكمال مهمة ما مستخداما التقنيات المساعدة اذا لم يكن بالجوار أي شخص يخبرني بماعلي فعله					
يمكنني اكمال مهمة ما مستخداما التقنيات المساعدة اذا كان بإمكاني الاتصال بشخص للمساعدة إذا واجهت مصاعب					
يمكنني اكمال مهمة ما مستخداما التقنيات المساعدة اذا كان لدي الكثير من الوقت لاكمالها					
يمكنني اكمال مهمة ما مستخداما التقنيات المساعدة اذا كان لدي فقط وسائل مدمجة للمساعدة					
سوف أكون قادر على النجاح في التغلب على الكثير من التحديات الدراسية باستخدام التقنيات المساعدة					
انا واثق من انني قادر على اؤدي بشكل فعال في العديد من المهام المختلفة باستخدام التقنيات المساعدة					
مقارنة مع الطلاب ضعاف البصر الاخرين الذين لا يستخدموا التقنيات المساعدة، يمكنني أن أفعل معظم المهام بشكل جيد.					

الجزء التاسع : القلق .

الرجاء اختيار ما يعبر عن مدى موافقتك على كل عبارة من العبارات التالية :

العنصر	لا أوافق بشدة	لا أوافق	محايد	أوافق	أوافق بشدة
أشعر بتخوف حول استخدام التقنيات المساعدة.					
إنه يخيفني التفكير في أنني يمكن أن أفقد الكثير من المعلومات أثناء استخدام التقنيات المساعدة بالضغط على الزر الخاطئ					
اتردد في استخدام التقنيات المساعدة خوفا من الوقوع في خطأ لا يمكن تصحيحه.					

التقنيات المساعدة تكون مخيفة بعض الشيء بالنسبة لي.					
سوف اكون ممانع لاستخدام التقنيات المساعدة لانني لست على دراية جيدة بها					

الجزء العاشر : إمكانية الوصول.

الرجاء اختيار ما يعبر عن مدى موافقتك على كل عبارة من العبارات التالية :

العنصر	لا أوافق بشدة	لا أوافق	محايد	أوافق	أوافق بشدة
لدي سهولة في الوصول إلى اجهزة التقنيات المساعدة في الجامعة.					
سهولة الوصول إلى أجهزة التقنيات المساعدة في العديد من المواقع في الجامعة ستكون مفيدة بالنسبة لي.					
توفير أجهزة التقنيات المساعدة في غرفة الصف مهم لنجاحي					
سهولة الوصول إلى أجهزة التقنيات المساعدة في المنزل والجامعة مفيد.					
اجهزة التقنيات المساعدة المحمولة والمتنقلة التي تحمل في كل مكان ستكون مفيدة					

الجزء العاشر : سلوك الاستخدام.

الرجاء اختيار ما يعبر عن مدى موافقتك على كل عبارة من العبارات التالية :

العنصر	لا أوافق بشدة	لا أوافق	محايد	أوافق	أوافق بشدة
اريد ان استخدم التقنيات المساعدة لاداء انشطتي الدراسية					
أنا أستخدم التقنيات المساعدة بشكل متكرر					
أنا أستخدم التقنيات المساعدة على نحو منتظم					
معظم مهامي الدراسية أنجزت باستخدام التقنيات المساعدة					

شكرا جزيلا على إعطاءنا من وقتك الثمين ومشاركتك في هذه الدراسة

- اذا كان لديك أية تعليق أو اقتراح الرجاء إضافته و كتابته في الأسطر التالية :

....
....
....
....
....
....
....

- إذا كنت مهتماً بنتائج البحث أو بالمشاركة مستقبلا في هذا الرجاء إضافة إيميلك
....
....
