# Supplementary material for: Assistive technology acceptance for visually impaired individuals: a case study of students in Saudi Arabia
Source: PeerJ Comput Sci. 2022 Mar 11;8:e886. doi: 10.7717/peerj-cs.886 (PMC9044340; doi:10.7717/peerj-cs.886)
Supplement: Supplemental Information 5 [file peerj-cs-08-886-s005.pdf]

أسئلة المقابلة

تسعى هذه المقابلة إلى التعرف على وجهة نظرك حول نتائج المسح البحثي المتعلق بمواقف الطلاب ذوي الإعاقة البصرية تجاه قبول واستخدام التقنية المساعدة. قبل الاشتراك في هذه المقابلة، الرجاء قراءة الوثيقة المعنونة "تقرير الاستبيان" والذي يوفر معلومات عن خلفية المشروع ومنهجية البحث ونتائج المسح البحثي.

معلوماتك

- في أي جامعة تدرس/تعمل ؟
- ماهو منصبك ان وجد؟
- كيف يمكنك وصف خبرتك مع التقنيات المساعدة لذوي الاعاقة البصرية؟
- ماهي أنواع التقنيات المساعدة التي تستعملها / تتعامل معها؟

وجهات نظرك

الفئة 1: الأداء المتوقع

- كيف يمكن أن يؤثر "توقع الأداء الجيد" في "نية استخدام التقنيات المساعدة"؟ لماذا ؟
- كيف تعتقد أن العمر سوف يؤثر على هذه العلاقة؟ يرجى توضيح؟
- كيف تعتقد أن الجنس يؤثر على هذه العلاقة؟ لماذا ا؟
- كيف تعتقد أن الخبرة في استخدام أجهزة الكمبيوتر سوف تؤثر على هذه العلاقة؟ لماذا ا؟
- كيف ترى أن مستوى التعليم سيؤثر على هذه العلاقة؟ يرجى توضيح؟
- كيف تعتقد أن مدة الإصابة بالإعاقة البصرية ستؤثر على هذه العلاقة؟ يرجى توضيح؟
- كيف تعتقد شدة الإصابة بالإعاقة البصرية ستؤثر على هذه العلاقة؟ يرجى توضيح؟

الفئة 2: الجهد المتوقع

- كيف يمكن أن يؤثر "توقع بذل جهد أقل عند استخدام التقنية المساعدة" في "نية استخدامها"؟ لماذا ؟
- كيف تعتقد أن العمر سوف يؤثر على هذه العلاقة؟ يرجى توضيح؟
- كيف تعتقد أن الجنس يؤثر على هذه العلاقة؟ لماذا ا؟
- كيف تعتقد أن الخبرة في استخدام أجهزة الكمبيوتر سوف تؤثر على هذه العلاقة؟ لماذا ا؟
- كيف ترى أن مستوى التعليم سيؤثر على هذه العلاقة؟ يرجى توضيح؟
- كيف تعتقد أن مدة الإصابة بالإعاقة البصرية ستؤثر على هذه العلاقة؟ يرجى توضيح؟
- كيف تعتقد شدة الإصابة بالإعاقة البصرية ستؤثر على هذه العلاقة؟ يرجى توضيح؟

الفئة 3: التاثير الإجتماعي

- كيف يمكن أن يؤثر "التأثير الإجتماعي" في "نية استخدام التقنيات المساعده" ؟ لماذا ؟
- كيف تعتقد أن العمر سوف يؤثر على هذه العلاقة؟ يرجى توضيح؟
- كيف تعتقد أن الجنس يؤثر على هذه العلاقة؟ لماذا ا؟
- كيف تعتقد أن الخبرة في استخدام أجهزة الكمبيوتر سوف تؤثر على هذه العلاقة؟ لماذا ا؟
- كيف ترى أن مستوى التعليم سيؤثر على هذه العلاقة؟ يرجى توضيح؟
- كيف تعتقد أن مدة الإصابة بالإعاقة البصرية ستؤثر على هذه العلاقة؟ يرجى توضيح؟
- كيف تعتقد شدة الإصابة بالإعاقة البصرية ستؤثر على هذه العلاقة؟ يرجى توضيح؟

الفئة 4: إمكانية الوصول

- كيف يمكن أن يؤثر " الدرجة التي يتمتع بها الشخص بالقدرة على الوصول إلى التقنيات المساعده " في "نية استخدامها" ؟ يرجى التوضيح ؟
- كيف تعتقد أن العمر سوف يؤثر على هذه العلاقة؟ يرجى توضيح؟
- كيف تعتقد أن الجنس يؤثر على هذه العلاقة؟ لماذا ا؟
- كيف تعتقد أن الخبرة في استخدام أجهزة الكمبيوتر سوف تؤثر على هذه العلاقة؟ لماذا ا؟
- كيف ترى أن مستوى التعليم سيؤثر على هذه العلاقة؟ يرجى توضيح؟
- كيف تعتقد أن مدة الإصابة بالإعاقة البصرية ستؤثر على هذه العلاقة؟ يرجى توضيح؟
- كيف تعتقد شدة الإصابة بالإعاقة البصرية ستؤثر على هذه العلاقة؟ يرجى توضيح؟

الفئة 5: الكفاءة الذاتية

- كيف يمكن أن تؤثر "زيادة قدرة الشخص على أداء مهمة معينة باستخدام التقنيات المساعده" في "نية استخدامه لها"؟ يرجى توضيح؟
- كيف تعتقد أن العمر سوف يؤثر على هذه العلاقة؟ يرجى توضيح؟
- كيف تعتقد أن الجنس يؤثر على هذه العلاقة؟ لماذا ا؟
- كيف تعتقد أن الخبرة في استخدام أجهزة الكمبيوتر سوف تؤثر على هذه العلاقة؟ لماذا ا؟
- كيف ترى أن مستوى التعليم سيؤثر على هذه العلاقة؟ يرجى توضيح؟
- كيف تعتقد أن مدة الإصابة بالإعاقة البصرية ستؤثر على هذه العلاقة؟ يرجى توضيح؟
- كيف تعتقد شدة الإصابة بالإعاقة البصرية ستؤثر على هذه العلاقة؟ يرجى توضيح؟

الفئة 6: القلق

- كيف يمكن "القلق المصاحب لاستخدام التقنيات المساعده" أن يؤثر على "نية المستخدم في استخدامها"؟ لماذا؟
- كيف تعتقد أن العمر سوف يؤثر على هذه العلاقة؟ يرجى توضيح؟
- كيف تعتقد أن الجنس يؤثر على هذه العلاقة؟ لماذا ا؟
- كيف تعتقد أن الخبرة في استخدام أجهزة الكمبيوتر سوف تؤثر على هذه العلاقة؟ لماذا ا؟
- كيف ترى أن مستوى التعليم سيؤثر على هذه العلاقة؟ يرجى توضيح؟
- كيف تعتقد أن مدة الإصابة بالإعاقة البصرية ستؤثر على هذه العلاقة؟ يرجى توضيح؟
- كيف تعتقد شدة الإصابة بالإعاقة البصرية ستؤثر على هذه العلاقة؟ يرجى توضيح؟

الفئة 7: الموقف تجاه استخدام التقنية

- كيف يمكن أن يؤثر "موقف المستخدم تجاه التقنية بشكل عام" على "النية السلوكية لاستخدام التقنيات المساعده"؟ يرجى توضيح؟
- كيف تعتقد أن العمر سوف يؤثر على هذه العلاقة؟ يرجى توضيح؟
- كيف تعتقد أن الجنس يؤثر على هذه العلاقة؟ لماذا ا؟
- كيف تعتقد أن الخبرة في استخدام أجهزة الكمبيوتر سوف تؤثر على هذه العلاقة؟ لماذا ا؟
- كيف ترى أن مستوى التعليم سيؤثر على هذه العلاقة؟ يرجى توضيح؟
- كيف تعتقد أن مدة الإصابة بالإعاقة البصرية ستؤثر على هذه العلاقة؟ يرجى توضيح؟
- كيف تعتقد شدة الإصابة بالإعاقة البصرية ستؤثر على هذه العلاقة؟ يرجى توضيح؟

الفئة 8: الظروف المساعدة

- كيف يمكن أن تؤثر "الظروف المساعدة مثل الحصول على الموارد والمعرفة اللازمة لاستخدام التقنيات المساعدة" على "الاستخدام الفعلي لهذه التقنيات"؟ يرجى توضيح؟
- كيف تعتقد أن العمر سوف يؤثر على هذه العلاقة؟ يرجى توضيح؟
- كيف تعتقد أن الجنس يؤثر على هذه العلاقة؟ لماذا ا؟
- كيف تعتقد أن الخبرة في استخدام أجهزة الكمبيوتر سوف تؤثر على هذه العلاقة؟ لماذا ا؟
- كيف ترى أن مستوى التعليم سيؤثر على هذه العلاقة؟ يرجى توضيح؟
- كيف تعتقد أن مدة الإصابة بالإعاقة البصرية ستؤثر على هذه العلاقة؟ يرجى توضيح؟
- كيف تعتقد شدة الإصابة بالإعاقة البصرية ستؤثر على هذه العلاقة؟ يرجى توضيح؟

الفئة 9: النية السلوكية لاستخدام التقنيات المساعده

- كيف يمكن أن تؤثر "نية استخدام التقنيات المساعده" على "سلوك المستخدم الفعلي"؟ لماذا؟

الفئة 10: معلومة اضافية

- يرجى اختيار الاكثر أهمية من العوامل التالية لتوضيح وجهة نظرك حول تأثيرها على قبول المستخدم للتقنيات المساعده.
  - (توقع الأداء)
  - (توقع الجهد)
  - (الموقف من استخدام التقنية)
  - (التأثير الإجتماعي)
  - (الظروف المساعدة)
  - (الكفاءة الذاتية)
  - (القلق)
  - (إمكانية الوصول للتقنية)
- لماذا تعتقد أن هذا العوامل مهمه؟
- لو أتيحت لك الفرصه لتطوير التقنيات المساعده , ماذا يتقترح ؟
- هل تعتقد أنه توجد عوامل أخرى قد تؤثر على قبول التقنية المساعدة؟
- هل لديك أي تعليقات أو ملاحظات؟

شكرا لك على الوقت الذي منحته للإجابة وعلى المساهمة القيمة في هذا البحث.
