# Supplementary material for: Assistive technology acceptance for visually impaired individuals: a case study of students in Saudi Arabia
Source: PeerJ Comput Sci. 2022 Mar 11;8:e886. doi: 10.7717/peerj-cs.886 (PMC9044340; doi:10.7717/peerj-cs.886)
Supplement: Supplemental Information 6 [file peerj-cs-08-886-s006.pdf]

Appendix A. Descriptive Statistics of the Observed Variables

Item	N	Minimum	Maximum	Mean	Std. Deviation	Skewness	Kurtosis
PE1	84	3.00	5.00	4.79	0.47	-2.11	3.89
PE2	84	2.00	5.00	4.68	0.56	-1.99	5.22
PE3	84	3.00	5.00	4.64	0.55	-1.25	0.64
PE4	84	1.00	5.00	4.39	0.78	-1.61	3.82
PE5	84	1.00	5.00	4.37	0.94	-1.78	3.18
PE6	84	1.00	5.00	4.24	0.83	-1.25	2.16
EE1	84	1.00	5.00	4.27	0.90	-1.91	4.76
EE2	84	1.00	5.00	4.23	0.80	-1.31	2.84
EE3	84	2.00	5.00	4.23	0.70	-0.56	0.03
EE4	84	1.00	5.00	4.23	0.84	-1.57	3.86
EE5	84	2.00	5.00	4.20	0.79	-0.83	0.41
EE6	84	2.00	5.00	4.20	0.72	-0.91	1.35
SI1	84	1.00	5.00	3.44	1.19	-0.30	-0.64
SI2	84	1.00	5.00	3.80	1.15	-0.86	0.10
SI3	84	1.00	5.00	2.87	1.29	-0.10	-1.09
SI4	84	1.00	5.00	3.12	1.35	-0.25	-1.20
SI5	84	1.00	5.00	2.61	1.41	0.23	-1.25
SI6	84	1.00	5.00	3.31	1.17	-0.54	-0.32
FC1	84	1.00	5.00	3.13	1.15	-0.16	-0.86
FC2	84	1.00	5.00	4.05	0.71	-1.10	3.60
FC3	84	1.00	5.00	3.46	0.97	-0.50	-0.01
FC4	84	1.00	5.00	2.98	1.46	-0.03	-1.33
FC5	84	1.00	5.00	3.89	0.86	-0.82	0.88
FC6	84	1.00	5.00	4.30	0.76	-1.24	2.93
ATT1	84	4.00	5.00	4.79	0.41	-1.42	0.01
ATT2	84	2.00	5.00	4.39	0.74	-1.15	1.04
ATT3	84	2.00	5.00	4.43	0.70	-1.04	0.67
ATT4	84	1.00	5.00	4.40	0.81	-1.58	3.12
ATT5	84	1.00	5.00	4.42	0.91	-1.92	3.97
ATT6	84	2.00	5.00	4.20	0.72	-0.72	0.56
BI1	84	2.00	5.00	4.39	0.66	-0.89	0.87
BI2	84	2.00	5.00	4.56	0.65	-1.46	2.16
BI3	84	2.00	5.00	4.42	0.79	-1.49	2.05

Item	N	Minimum	Maximum	Mean	Std. Deviation	Skewness	Kurtosis
BI4	84	1.00	5.00	4.46	0.81	-1.88	4.18
BI5	84	2.00	5.00	4.55	0.68	-1.68	3.17
SE1	84	2.00	5.00	4.08	0.85	-0.76	0.10
SE2	84	1.00	5.00	4.08	0.82	-1.08	1.91
SE3	84	1.00	5.00	4.07	0.90	-1.25	2.11
SE4	84	1.00	5.00	3.55	0.96	-0.39	-0.12
SE5	84	2.00	5.00	4.56	0.59	-1.33	2.72
SE6	84	3.00	5.00	4.63	0.51	-0.83	-0.68
SE7	84	1.00	5.00	4.27	0.95	-1.71	3.35
AN1	84	1.00	5.00	1.77	1.05	1.38	1.21
AN2	84	1.00	5.00	2.63	1.25	0.17	-1.26
AN3	84	1.00	5.00	1.95	1.04	1.08	0.60
AN4	84	1.00	5.00	1.94	1.19	1.23	0.49
AN5	84	1.00	5.00	1.52	0.86	1.92	3.80
AC1	84	1.00	5.00	2.85	1.31	-0.13	-1.12
AC2	84	1.00	5.00	4.48	0.81	-1.91	4.28
AC3	84	2.00	5.00	4.46	0.78	-1.50	1.83
AC4	84	3.00	5.00	4.71	0.48	-1.30	0.48
AC5	84	4.00	5.00	4.82	0.39	-1.71	0.94
UB1	84	2.00	5.00	4.56	0.72	-1.92	3.98
UB2	84	2.00	5.00	4.45	0.72	-1.53	2.87
UB3	84	2.00	5.00	4.24	0.87	-1.04	0.45
UB4	84	1.00	5.00	4.26	0.92	-1.31	1.47
